# Plant-derived extracellular vesicles in skin and bone tissue engineering: current status, challenges, and future perspectives

**DOI:** 10.3389/fbioe.2026.1764724

**Published:** 2026-03-19

**Authors:** Chenyang Lu, Dipeng Li, Wenxin Gao, Xiaoxian Sun, Yan Zhang, Yunfei Yu, Chenghao Fei, Peina Zhou, Mao Wu

**Affiliations:** 1 Department of Trauma and Orthopedics, Wuxi Affiliated Hospital of Nanjing University of Chinese Medicine, Wuxi, China; 2 Hangzhou Ninth Hospital, Hangzhou, China; 3 The First Affiliated Hospital, Zhejiang Chinese Medical University, Hangzhou, Zhejiang, China; 4 College of Horticulture, Nanjing Agricultural University, Nanjing, China; 5 Nanjing Research Institute for Comprehensive Utilization of Wild Plants, All China Federation of Supply and Marketing Cooperatives, Nanjing, China

**Keywords:** drug delivery, plant-derived extracellular vesicles, plant-derived nanovesicles, regenerative medicine, tissue engineering

## Abstract

Plant-derived extracellular vesicles (PDEVs) are bioactive nanoscale vesicles secreted by plant cells, which have recently gained attention as promising therapeutic agents in tissue engineering owing to their low immunogenicity, inherent biological activities, and potential as drug delivery vehicles. This review comprehensively outlines the general properties, application-specific characteristics, and isolation techniques of PDEVs, with a particular emphasis on their roles in facilitating cell proliferation, differentiation, and immunomodulation to promote tissue regeneration. We further discuss the therapeutic efficacy of PDEVs derived from various plant sources across different tissue engineering contexts, as well as the application of engineered PDEVs in tailored regenerative strategies. In comparison to mammalian extracellular vesicles, PDEVs present distinct advantages, including minimized ethical concerns and reduced risks of immune rejection. Nevertheless, challenges remain for their clinical translation, such as the lack of standardized isolation protocols and inadequate assessment of long-term *in vivo* safety. This article synthesizes current understanding of PDEVs, underscores their multifunctional potential, and offers perspectives on engineering approaches aimed at enhancing their therapeutic performance. With continued development, PDEVs may emerge as innovative tools in tissue engineering, facilitating tissue repair and regeneration either through their innate bioactivity or as engineered drug delivery systems.

## 1 Introduction

Extracellular vesicles (EVs) are nanoscale membrane-bound structures released by cells that carry a diverse cargo of biomolecules, including proteins, lipids, RNAs, metabolites, growth factors, and cytokines (Abels and Breakefield, 2016). Serving as critical mediators of intercellular communication, EVs play essential roles in a wide range of physiological and pathological processes. These vesicles facilitate the transfer and regulatory functions of bioactive molecules, including proteins, lipids, nucleic acids, and small molecules of secondary metabolism, between cells (Couch et al., 2021). Based on their biogenesis, physical properties, and biochemical composition, EVs are commonly classified into three main subtypes: exosomes (30–150 nm), which originate from the endosomal pathway; microvesicles (MVs, 50–1,000 nm), which are shed directly from the plasma membrane; and apoptotic bodies (50–5,000 nm), which are released during programmed cell death (van Niel et al., 2018; Zhang et al., 2020). Animal-derived EVs have been extensively studied due to their critical functions in various fields, including cancer, musculoskeletal disorders, skin wounds, neurological diseases, and cardiovascular diseases (Bai et al., 2024; Kobayashi-Sun et al., 2020; O’Brien et al., 2020). With their potent biological activity and intercellular communication capabilities, EVs demonstrate significant potential as therapeutic tools. Growing evidence indicates that exosomes can serve as functional carriers, contributing therapeutic value in regenerative medicine by modulating intercellular communication, immune homeostasis, and tissue repair processes. As the field of exosome research continues to expand, plant-derived extracellular vesicles (PDEVs) have garnered extensive research interest. With inherent advantages such as biosafety and low production costs, PDEVs not only complement animal-derived exosomes but also represent a highly promising class of natural nanocarriers.

PDEVs are nano- to micro-scale membrane vesicles (50–1,000 nm) released by plant cells, which are biogenically and morphologically comparable to mammalian extracellular vesicles (An et al., 2006). Commonly referred to in the literature as plant exosome-like nanovesicles or edible plant-derived nanovesicles, they constitute a heterogeneous population originating from various cellular compartments, including polyvesicular bodies, autophagosomes, vacuoles, and exocyst-positive organelles (EXPO). PDEVs carry a variety of functional biomolecules, including proteins, miRNAs, lipids, nucleic acids, and small-molecule compounds. Their lipid profiles are predominantly composed of phosphatidic acid, phosphatidylcholine, digalactosyldiacylglycerol, monogalactosyldiacylglycerol, and phytosterols (Liu et al., 2020). The study of PDEVs traces back to 1967, when Halperin and colleagues first observed vesicular structures released into the extracellular space from carrot cell cultures, suggesting their role in cell wall remodeling and cell separation (Halperin and Jensen, 1967). Since then, research on PDEVs has expanded considerably, laying a solid foundation for their application in biomedical fields. PDEVs offer several compelling advantages that make them promising candidates for drug delivery and tissue engineering applications. PDEVs exhibit high safety and low immunogenicity. Kim J. et al. (2022) demonstrated that grapefruit-derived PDEVs (GDNVs) possess outstanding *in vivo* safety, with low organ accumulation and minimal systemic toxicity. PDEVs enable cost-effective, scalable production. They can be extracted at low cost from abundant edible plants, facilitating large-scale manufacturing and sustainable supply (Alzahrani et al., 2023). PDEVs enable efficient cross-species delivery. Their high biocompatibility allows effective penetration of biological barriers for biodistribution, facilitating cross-species communication and drug delivery (Stotz et al., 2022). PDEVs possess inherent biological activity: Certain PDEVs enhance therapeutic potential through intrinsic pharmacological activity mediated by biomolecules such as miRNAs and lipids. These properties have spurred growing interest in applying PDEVs in tissue engineering—a core discipline within regenerative medicine that aims to repair or regenerate damaged tissues using cells, biomaterial scaffolds, and bioactive molecules (Luo et al., 2019). It has shown significant clinical potential across various domains, including skin, cartilage, bone, and nerve regeneration (Zhu et al., 2025). Recent studies highlight their potential across various tissue contexts. EVs derived from aloe vera, neem, and ginger, incorporated into chitosan–polyvinyl alcohol nanofilms, facilitate sustained release that modulates inflammation and oxidative stress, significantly enhancing wound healing in diabetic rats (Miya et al., 2025). Spirulina-derived PDEVs (SP-EVs), delivered via rhein-based hydrogel, regulate energy homeostasis and suppress inflammation through the IL-6/JAK2/STAT3 pathway, promoting articular cartilage repair (Liang et al., 2025). Cissus quadrangularis-derived PDEVs stimulate osteogenic differentiation of mesenchymal stem cells and accelerate wound healing (Gupta et al., 2023). Lycium barbarum-derived vesicles loaded with isoliquiritin within 3D scaffolds promote functional recovery in spinal cord injury models via activation of the pAKT pathway and M2 macrophage polarization, demonstrating their ability to cross the blood-spinal cord barrier (Wang et al., 2024). Despite these promising results in preclinical models, current research on PDEVs remains largely experimental. Clinical evidence is still limited, underscoring the need for further mechanistic studies and standardized protocols to advance their translational potential in tissue engineering and regenerative medicine.

Unlike previous reviews that primarily focus on PDEVs as general drug delivery vectors or their role in cancer therapy, this article specifically centers on the unique requirements of structural tissue repair—namely, skin and bone. Currently, chronic wounds and bone defects remain difficult clinical challenges with limited effective solutions. While EVs are a mature field of study, PDEVs have emerged as a distinct research hotspot in recent years due to their natural advantages. By synthesizing current findings on PDEV isolation, characterization, and engineering, this review aims to offer practical suggestions and guidance to assist researchers in identifying future directions in this field.

## 2 General characteristics of PDEV

### 2.1 Naming and classification of PDEV

According to the guidelines established by the International Society for Extracellular Vesicles (ISEV), the term “extracellular vesicles” (EVs) is recommended as the standardized nomenclature to describe naturally secreted particles that are delimited by a lipid bilayer and lack the capacity for self-replication (Witwer and Thery, 2019).

ISEV recommends classifying EVs into two main subtypes based on physical diameter: small extracellular vesicles (sEVs), typically ranging from 40 to 150 nm, and large extracellular vesicles (lEVs), which generally span from 200 nm to 10 μm. It is important to note that some overlap may exist in the size ranges defined by different biogenesis pathways (Mariscal et al., 2020). These EVs, which transport a diverse array of biomolecules, can also be subclassified according to their biogenesis pathways (Shah et al., 2018). Mammalian EVs exhibit considerable heterogeneity and have conventionally been classified—based on their biosynthetic mechanisms—into exosomes, microvesicles, apoptotic bodies, and other subtypes (Anand et al., 2019). Similarly, plant-derived vesicle-like nanoparticles (PDVNs) display significant heterogeneity in both cellular origin and physical characteristics.

Currently, PDEVs lack a standardized nomenclature system and well-defined classification criteria. Commonly used terms include “extracellular vesicles,” “extracellular nanovesicles,” and “extracellular vesicle-like nanoparticles.” In experimental practice, the precise categorization of the isolated vesicles remains challenging due to variations in plant material processing and extraction protocols. To facilitate consistency in scientific reporting, Pinedo et al. (2021) recommend using the general term “plant EVs” rather than “exosomes” until the specific characteristics of plant exosomes are fully elucidated and established as a basis for classification. Moreover, when vesicles are obtained using destructive extraction methods or do not represent naturally secreted structures, the resulting isolates should be referred to as “plant-derived nanovesicles (PDNV)”. To ensure consistency, we will use the general term PDEV and, for the time being, set aside definitional discrepancies related to extraction methods. Any vesicles requiring specific distinction or those that are unidentifiable will be named according to the source literature or uniformly explained as PDEV.

### 2.2 Pathways of PDEV occurrence

**FIGURE 1:**
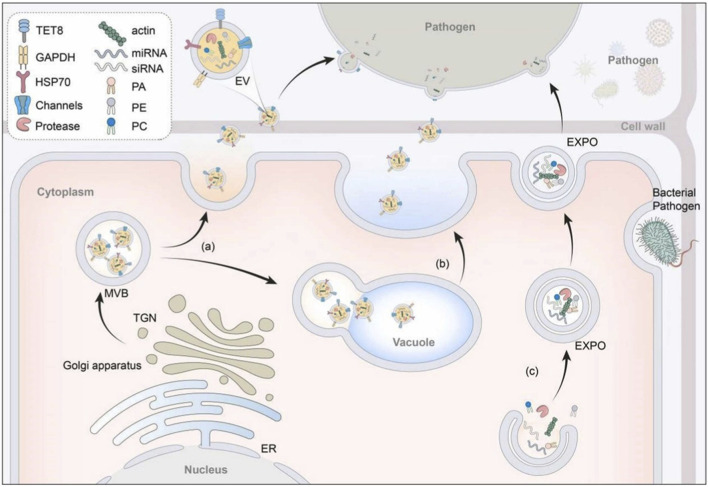
Taking exosomes as an example, elucidating the basic morphology, constituents, and release mechanisms of PDEV in plants (Mao et al., 2024). Plant cells excrete EVs to counter bacterial, fungal, and other pathogenic invasions through the following pathways. Pathway **(a)**. delineates the fusion of MVB and the plasma membrane, resulting in the release of intraluminal vesicles (ILVs) as exosomes. Pathway **(b)**. describes the release of intraluminal EV (IEV) through the vacuolar fusion with plasma membranes. Pathway **(c)**. shows EXPO secretion. Abbreviations: MVB, multivesicular bodies; ER, endoplasmic reticulum; TNG, trans-Golgi network; EXPO, exocyst positive organelles. Abbreviations: MVB, multivesicular body; EXPO, exocyst-positive organelle; sRNA, small RNA; mRNA, messenger RNA.

Currently, three main biogenesis pathways have been identified for PDEVs: the multivesicular body (MVB) pathway, the EXPO pathway, and the vacuolar pathway (As shown in Figure 1) (Mao et al., 2024).

The MVB pathway represents the primary mechanism for the secretion of EVs by plant cells. MVBs originate from early endosomes and contain numerous intraluminal vesicles (ILVs) within their lumen. Upon fusion of MVBs with the plasma membrane, these ILVs are released into the extracellular space, thereby forming PDEVs (Halperin and Jensen, 1967). As early as the 1960s, researchers observed the fusion of MVBs with the plasma membrane and the subsequent release of secondary vesicles in carrot cell cultures. This biogenetic pathway is particularly activated during plant immune responses against pathogens. For instance, in *Arabidopsis thaliana* infected with turnip mosaic virus (TuMV), MVBs proliferate extensively, fuse with the plasma membrane, and release vesicles that carry viral RNA (Movahed et al., 2019).

The EXPO pathway represents an unconventional secretory mechanism unique to plants. EXPOs are spherical, double-membrane-delimited organelles that originate from the trans-Golgi network (TGN). These organelles undergo direct fusion with the plasma membrane, thereby releasing single-membrane vesicles into the extracellular space. This pathway contributes to intercellular communication under normal physiological conditions, a role that has been clearly documented in *Arabidopsis thaliana* (Jadli et al., 2020).

In addition to the MVB and EXPO pathways, the vacuolar pathway has been implicated in the formation of plant EVs. This mechanism involves the fusion of either central vacuoles or small vacuoles (SVs) with the plasma membrane, leading to the release of vesicular contents. These small vacuoles, which mature from MVBs, fuse with the plasma membrane and release their internal intraluminal vesicles (IEVs) into the extracellular space (Li Y. et al., 2024). Under pathogenic challenge, vacuoles containing hydrolases and defense-related compounds fuse with the plasma membrane, facilitating the extracellular release of these components to inhibit pathogen proliferation (Hatsugai et al., 2009). This pathway plays an essential role in plant immunity. For instance, *Arabidopsis thaliana* secretes antibacterial proteins via the vacuolar route to induce pathogen death (Cui et al., 2019).

### 2.3 Characterization of PDEV

#### 2.3.1 Physical characterization of PDEV

Physical characterization of PDEVs typically includes assessment of morphology, concentration, size distribution, and zeta potential. Morphological analysis is critical for elucidating the structural attributes of PDEVs. Techniques such as transmission electron microscopy (TEM) and scanning electron microscopy (SEM) are widely used to visualize these vesicles, revealing a diversity of shapes, including spherical, cup-shaped, tubular, and irregular forms. A comprehensive understanding of PDEVs morphology is essential for inferring their biological functions and mechanisms of interaction with target cells or tissues (Yang et al., 2025; Gandham et al., 2020).

Atomic force microscopy (AFM), dynamic light scattering (DLS), and nanoparticle tracking analysis (NTA) are routinely used to determine the size distribution and concentration of PDEVs. These particles generally exhibit diameters ranging from tens to hundreds of nanometers, with precise dimensions influenced by the plant source and isolation methodology (Yuana et al., 2010; Labrie et al., 2013; Dragovic et al., 2011). Accurate characterization of size and concentration is essential for standardizing PDEVs preparations and optimizing their efficacy in biomedical applications, such as drug delivery and diagnostic development.

The zeta potential of PDEVs exhibits considerable variation across different plant species, generally ranging from approximately −1.5 to −49.2 mV, with the majority of PDEVs displaying a net negative surface charge (Zhu et al., 2023; Seo et al., 2023; Yoon et al., 2024).

#### 2.3.2 Biochemical characterization of PDEV

**FIGURE 2:**
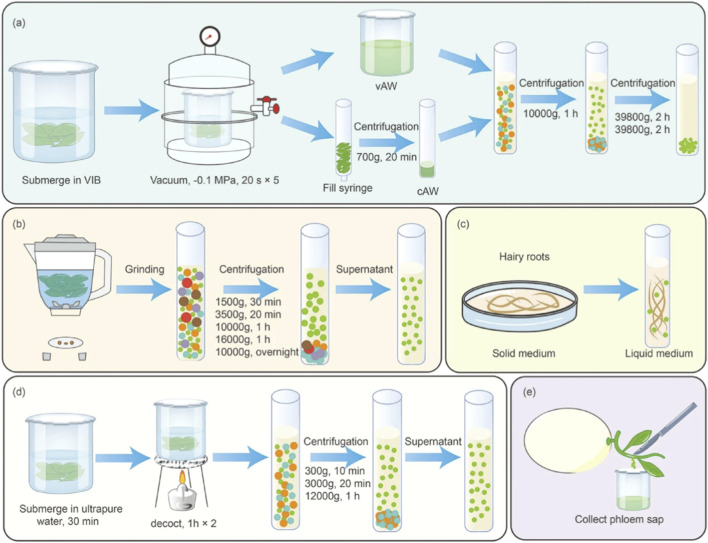
Schematic illustration of common preprocessing methods for the isolation of PDEVs (Pulido-Escribano et al., 2025). **(a)** Tissue infiltration centrifugation; **(b)** Tissue disruption; **(c)** Conditioned medium method; **(d)** Decoction method; **(e)** Stem incision method. Abbreviations: VIB, vesicle isolation buffer; vAW, vacuum-assisted washing; cAW, centrifugation-based washing.

Multimodal omics approaches have become instrumental in the systematic identification and functional characterization of cargo molecules within EVs and PDEVs (Wang F. et al., 2025). To elucidate their biochemical composition, a range of analytical techniques—such as Western blotting, mass spectrometry, ELISA, and gel electrophoresis—are routinely employed. PDEVs are nanoscale lipid bilayer-enclosed structures that carry a diverse cargo of biomolecules—including lipids, proteins, nucleic acids, and metabolites—within their lumen (As shown in Figure 2) (Anand et al., 2019).

Lipids serve as the fundamental structural elements of the bilayer membrane in PDEVs. These lipid components can be characterized using analytical techniques such as thin-layer chromatography (TLC), mass spectrometry (MS), and high-performance liquid chromatography (HPLC). Meanwhile, the overall protein profile can be assessed through SDS-polyacrylamide gel electrophoresis (SDS-PAGE). Notably, Zeng et al. (2021) identified glucosylceramide (GlcCer) as the predominant lipid in aloe-derived PDEVs (ADNVs), revealing its critical role as a driver of membrane curvature during exosome biogenesis and secretion. This finding further supports the existence of a natural and evolutionarily conserved secretory mechanism in ADNVs.

PDEVs commonly contain cytoplasmic proteins, membrane channel proteins, and specific surface marker proteins. Their proteomic profiles vary significantly depending on the plant source and the biogenesis pathway involved. Martínez-Ballesta et al. (Sriwastva et al., 2022) identified heat shock proteins HSPA8 and HSP70 in mulberry bark-derived EVs and demonstrated their protective effect against sodium dextran sulfate (DSS)-induced colitis. Using LC-MS/MS proteomics, Sánchez-López et al. (2022) detected 131 proteins in pomegranate-derived PDEVs (PgEVs), several of which are implicated in EV biogenesis and transport, suggesting their potential use as specific markers for PgEVs. However, a critical distinction must be made regarding protein characterization. Standard markers used for mammalian EVs (e.g., CD9, CD63, CD81) are generally inapplicable to plant vesicles due to the lack of homologous proteins in plants. Researchers must exercise caution when using antibodies targeting human or murine proteins, as low sequence homology and distinct glycosylation patterns in plant proteins can lead to non-specific binding and misleading false-positive results. Consequently, the field is moving away from relying on mammalian antibodies and towards verifying plant-specific markers (such as TET8 in Arabidopsis) identified through rigorous proteomic analysis. In another study, De Palma et al. (2020) reported that the membrane protein p34 in tomato-derived EVs participates in reactive oxygen species (ROS) signaling and vesicular transport. Additionally, aquaporin family members, particularly major intrinsic proteins (MIPs), were identified in tomato EVs; these proteins form channels that facilitate the transport of water, small neutral solutes, and gases (Kapilan et al., 2018).

sRNA sequencing has revealed the presence of numerous sRNAs within PDEVs. These sRNAs have been well-established as key regulators of critical biological processes in both plants and animals, modulating mechanisms such as cell differentiation, programmed cell death, proliferation, immune responses, normal growth and development, as well as adaptations to biotic and abiotic stresses (Lopez de Las Hazas et al., 2023).

In addition to lipids, proteins, and small RNAs, certain PDEVs also encapsulate a variety of small-molecule compounds. These molecules often correspond to secondary metabolites that contribute to the intrinsic therapeutic properties of PDEVs (Zhang et al., 2016). For example, Zhang et al. identified the presence of 6-gingerol and 6-shogaol—key bioactive constituents of ginger—within ginger-derived PDEVs, suggesting their potential role in mediating the anti-inflammatory effects associated with these vesicles (Bischoff-Kont and Fürst, 2021; Hong et al., 2020). Similarly, Wang et al. (2014) confirmed the enrichment of polyphenolic compounds such as naringin and naringenin in grapefruit-derived PDEVs, underscoring the potential contribution of these metabolites to the observed pharmacological activities (Zeng et al., 2018).

### 2.4 Application characteristics of PDEV

#### 2.4.1 Stability

PDEVs exhibit remarkable stability, which is essential for their potential biomedical applications. The zeta potential serves as a key indicator for evaluating the colloidal stability of nanoparticles, with higher absolute values generally correlating with greater system stability. Most PDEVs in aqueous solutions display zeta potentials within the range of −30 mV to +30 mV, consistent with stable colloidal systems and indicative of favorable dispersibility and storage stability (Bommakanti et al., 2022; Ou et al., 2023). Another crucial criterion for assessing the applicability of biomaterials is their stability across varying pH conditions (Zhang et al., 2023). Yang et al. (2025) demonstrated that PDEVs derived from Platycodon grandiflorum (PGEVs) retained their structural integrity in the highly acidic environment of the gastrointestinal tract (GIT) and resisted enzymatic degradation for over 24 h. Similarly, Ou et al. (2023) confirmed that PDEVs (CLDENs) isolated from Catharanthus roseus (L.) Don leaves maintained intact membrane structures under both acidic and alkaline conditions without observable vesicle rupture, underscoring the robust acid-base resistance of PDEVs.

#### 2.4.2 Biocompatibility

PDEVs exhibit remarkable biocompatibility, a key advantage for their therapeutic potential. Structurally and biogenetically, PDEVs share significant similarities with mammalian-derived EVs. Like EVs of human origin, PDEVs can be internalized by recipient cells via plasma membrane fusion, a mechanism that facilitates their traversal across physiological barriers—including the blood–brain barrier. For instance, Kim et al. (2023a) successfully isolated ginseng-derived PDEVs (GENs) and demonstrated their ability to cross the blood–brain barrier, thereby inhibiting glioma progression through modulation of the tumor microenvironment. Moreover, PDEVs generally exhibit a superior safety profile compared to synthetic nanoparticles. However, their ‘low immunogenicity’ should be interpreted as immune tolerance rather than complete invisibility. PDEVs possess distinct surface signatures, particularly plant-specific glycosylation motifs (such as high-mannose, β1,2-xylose, and α1,3-fucose). These motifs facilitate specific recognition by the innate immune system, thereby promoting efficient cellular uptake and subsequent immunomodulatory effects without triggering severe acute rejection (Logozzi et al., 2022). Zhang et al. (Wang et al., 2013) proposed that this biocompatibility may arise from the activation of the AMPK signaling pathway in dendritic cells, leading to an immunotolerant response.

#### 2.4.3 Security

Clinical studies have demonstrated that certain formulations of PDEVs exhibit a highly favorable safety profile, as supported by numerous *in vitro* and *in vivo* experiments (Kim et al., 2023a; Feng et al., 2023). This biocompatibility may be attributed to the fact that PDEVs are often sourced from edible or medicinal plants with established histories of clinical use. As a result, these vesicles are generally well-tolerated by the human immune system. Nevertheless, the dual nature of PDEV immunogenicity—being generally safe for tissue repair while potent enough to act as vaccine adjuvants when engineered—highlights the complexity of their interaction with the host immune system. The potential for plant-derived proteins or glycans to act as allergens in specific populations cannot be entirely ruled out. Therefore, while current data suggests a reduction in immunogenic risks compared to animal-derived EVs, rigorous immunological profiling is essential for clinical translation (Logozzi et al., 2022; Pulido-Escribano et al., 2025; Zhang et al., 2025; Kürtösi et al., 2024). Furthermore, repeated oral administration of PDEVs has been shown not to alter physiological, biochemical, or behavioral parameters in experimental animals, and does not elicit local or systemic toxic reactions (Zhang et al., 2016). Importantly, engineered PDEV-based systems retain this favorable safety profile while exhibiting enhanced therapeutic efficacy. For example, Wang et al. (2013) developed nanoparticles (GNVs) using lipids derived from grapefruit PDEVs, which were further functionalized with folic acid to construct a versatile delivery platform. These engineered GNVs efficiently delivered chemotherapeutic agents, siRNA, DNA expression vectors, and proteins to diverse cell types. In contrast to synthetic lipid-based nanoparticles, GNVs demonstrated reduced cytotoxicity. Following intravenous administration in pregnant mice, GNVs did not traverse the placental barrier, induce elevated inflammatory cytokine levels or liver enzyme elevations, or cause any histopathological abnormalities—collectively affirming their preclinical safety.

#### 2.4.4 Low production costs

Although current research on PDEVs remains less advanced than that on mammalian EVs, PDEVs have emerged as a highly promising alternative therapeutic platform, owing to their significant economic and scalability advantages. Plant sources—such as citrus, ginger, and broccoli—are widely available and inexpensive, with production costs potentially as low as one-tenth of those required for mammalian cell culture. Moreover, plant tissues typically yield substantially higher quantities of EVs compared to mammalian systems, dramatically lowering the expenses associated with large-scale manufacturing (Logozzi et al., 2022).

#### 2.4.5 Limitations

A major current research priority and challenge in clinical translation is the development of large-scale, cost-effective, and highly reproducible production processes for PDEVs. The production of PDEVs is constrained by seasonal and regional variations inherent in plant agriculture. Long-term field cultivation is not only time-consuming and labor-intensive but also complicated by significant interspecies differences in growth cycles. Moreover, the distinct biological structure of plants necessitates specialized isolation methods, as techniques directly adapted from mammalian EV protocols are often unsuitable. Raw material quality presents another critical hurdle. Plants grown in open environments are vulnerable to viral contamination, and the similar sizes of viral particles and PDEVs substantially complicate downstream separation and purification, demanding more advanced technical approaches. Additionally, current protocols predominantly rely on fresh plant materials, which imposes stringent requirements on harvesting, preservation, and storage conditions for maintaining PDEVs integrity and function.

## 3 Isolation, extraction, and storage methods for PDEV

### 3.1 Preprocessing for PDEV

Pretreatment of plant tissues constitutes the initial and critical step in the isolation and purification of PDEVs. The most widely used pretreatment approaches include tissue immersion and physical disruption. Additionally, several specialized techniques have been developed for specific applications (As shown in Figure 2) (Liu et al., 2025).

Tissue immersion involves incubating plant samples in permeabilization buffers to facilitate the extraction of apoplastic fluid. This method helps maintain cellular integrity and is commonly applied to leaf tissues. For instance, in *Arabidopsis thaliana* leaves, vacuum infiltration is frequently employed: tissues are immersed in buffer and subjected to three cycles of vacuum pulses (10 s each, with 30-s intervals), followed by low-speed centrifugation to remove cellular debris. The resulting supernatant, known as apoplastic washing fluid (AWF), is enriched with EVs and is suitable for obtaining high-purity isolates (Rutter and Innes, 2017). However, this method presents certain limitations. The immersion buffer may dilute exosomal concentrations and elicit plant stress responses, stimulating the secretion of metabolites that can contaminate or reduce the yield of PDEVs. Consequently, its application has so far been confined mainly to leaf tissues, with no successful reports yet in roots, stems, or fruits.

Tissue disruption methods typically involve mechanical homogenization of plant materials to obtain a crude extract, which is then subjected to low-speed centrifugation to remove large fibrous particles and cellular debris. Most protocols incorporate two to three rounds of low-speed centrifugation to eliminate residual cellular and organellar contaminants, followed by ultracentrifugation to enrich PDEVs. The resulting vesicle pellets are commonly resuspended in phosphate-buffered saline (PBS) (Perut et al., 2021; Choi et al., 2024; Gong et al., 2025). This approach yields substantially higher PDEVs concentrations compared to tissue immersion methods. Although mechanical disruption enhances yield, it inevitably causes cellular damage, which can lead to the fusion of organellar or plasma membrane fragments with genuine EVs. Proteomic analyses reveal that EV samples prepared this way often contain membrane fragments and intracellular proteins—such as those derived from the cytoplasm, nucleus, and other organelles—resulting from cell lysis (Perut et al., 2021). Therefore, it is essential to establish standardized operating procedures that control variables including plant source, grinding intensity, duration, and centrifugation parameters to minimize artifacts and reduce heterogeneity in the biochemical composition of isolated PDEVs.

In addition to conventional approaches, several specialized pretreatment methods have been developed for the isolation of PDEVs. Among these, the decoction method utilizes boiling water as a solvent to extract bioactive components. Li et al. (Gao Y. et al., 2022; Li T. et al., 2024; Li et al., 2019) applied this technique to American ginseng and demonstrated that the resulting PDEVs exhibited enhanced therapeutic efficacy. Alternatively, Vestuto et al. (2024) employed a biological cultivation strategy by establishing sage hairy root cultures, collecting the conditioned medium, and subsequently isolating PDEVs through ultracentrifugation or size exclusion chromatography. In a different approach, Sánchez-López et al. (2024) collected phloem sap from melon plants via stem incision and successfully isolated melon-derived vesicles (ML-DVs) using size exclusion chromatography.

### 3.2 Isolation and extraction of PDEV

**FIGURE 3:**
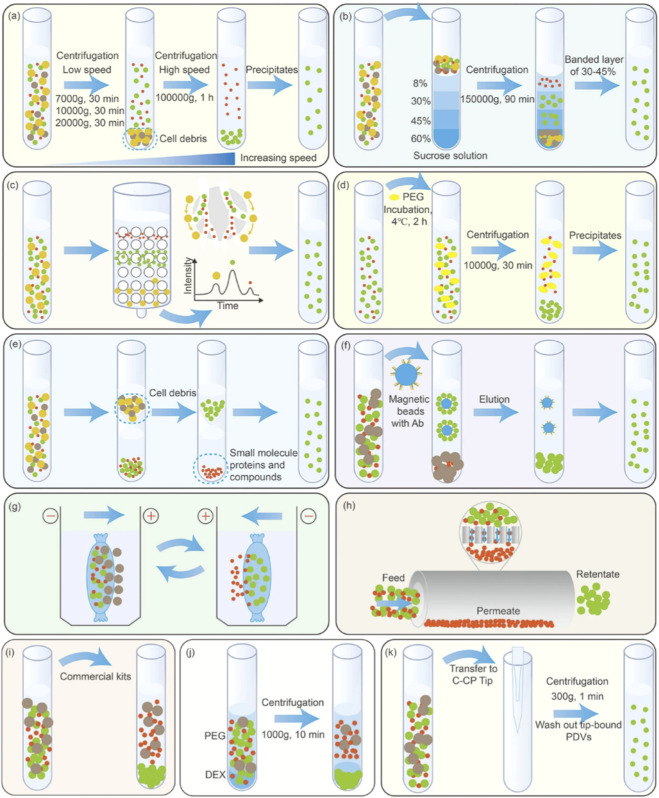
Methods for the Separation of PDEVs.Illustrated are commonly employed techniques for PDEV isolation (Pulido-Escribano et al., 2025): **(a)** Differential ultracentrifugation; **(b)** Density gradient centrifugation; **(c)** Size exclusion chromatography; **(d)** Polyethylene glycol (PEG)-based precipitation; **(e)** Ultrafiltration; **(f)** Immunoaffinity capture; **(g)** Electrophoresis; **(h)** Tangential flow filtration; **(i)** Commercial kit-based isolation; **(j)** Aqueous two-phase separation; **(k)** Capillary channel polymer (C-CP) fiber spin-tip method.

A variety of methodologies are available for the separation and extraction of PDEVs (As shown in Figure 3) (Liu et al., 2025). The choice of separation technique significantly impacts the purity, yield, and biological activity of the resulting PDEVs preparations. As shown in Figure 3, the choice of separation method—from traditional ultracentrifugation (A) to emerging aqueous two-phase separation (J)—requires balancing yield, purity, and operational complexity. For a detailed comparison of each separation technique, see Table 1. Table 1 summarizes commonly employed PDEVs isolation technologies, comparing the advantages and limitations of each method, along with their suitable plant sources.

**TABLE 1:** Techniques for the separation and purification of PDEV and their applications.

Separation technique	Principle	Key parameters (Typical yield, purity, processing time)	Advantages and disadvantages	Suitable scenarios
Differential centrifugation (DC)	Density	Yield: Medium; Purity: Low-Medium; Processing Time: 2–4 h (for one round)	Advantages: Handles large volumes; no requirement for specialized additives. Disadvantages: Potential vesicle damage; time-consuming; equipment-intensive	Large-scale preparation; Initial enrichment step
Density gradient centrifugation (DGC)	Precipitation velocity and density magnitude	Yield: Medium; Purity: High; Processing Time: 16–20 h (including gradient formation)	Advantages: High purity and resolution; minimal mechanical damage. Disadvantages: Lengthy procedure; gradient medium may contaminate samples	Basic research requiring high purity; Small-scale precise isolation
Ultrafiltration centrifugation (UFC)	Particle size	Yield: High; Purity: Medium; Processing Time: 1–3 h	Advantages: High efficiency; low cost; preserves native morphology. Disadvantages: Membrane fouling; protein contamination	Medium to large-scale preparation; Rapid initial concentration
Size exclusive chromatography (SEC)	Particle size	Yield: Medium-High; Purity: High; Processing Time: 1–2 h (per run, column reusable)	Advantages: Good purity; gentle process; reusable columns. Disadvantages: Limited sample volume per run; dilution of samples	Small to medium-scale preparation; Applications requiring high biological activity
Polymer-based precipitation	Reduce the Solubility of EV in Water	Yield: High; Purity: Low; Processing Time: 4–12 h (including incubation)	Advantages: Simple; scalable; no special equipment. Disadvantages: Low purity; co-precipitation of contaminants	Large-scale initial capture; Pre-purification for diagnostic use
Immunoaffinity capture-based technique	Directional Enrichment of EV with Specific Covalent or Affinity Magnetic Beads	Yield: Low; Purity: Very High; Processing Time: 2–6 h (highly variable)	Advantages: Extremely high specificity and purity. Disadvantages: Low yield; high cost; limited to known markers	Target-specific basic research; Small-scale studies requiring highly specific subpopulations
Field flow fractionation (AF4)	A force field is applied perpendicular to the sample flow, separating particles based on the differences in diffusion coefficients between particles of varying sizes	Yield: Medium; Purity: High; Processing Time: 1–2 h	Advantages: High resolution; minimal shear stress; no stationary phase. Disadvantages: Specialized equipment; optimization required	Basic research; Characterization and functional analysis of subsets
ATPS	Utilizing phase systems formed by two immiscible polymers or polymer-salt solutions for partitioning separation	Yield: Medium; Purity: Medium-High; Processing Time: 3–8 h	Advantages: Gentle conditions; effective contaminant removal. Disadvantages: System optimization can be complex	Medium-scale preparation; Purification of sensitive vesicles

#### 3.2.1 Ultracentrifugation, UC

Ultracentrifugation (UC) serves as a foundational technique for the separation and purification of PDEVs, employing principles analogous to those used in animal EV isolation (Gardiner et al., 2016). Typical protocols involve an initial low-speed centrifugation step (500–10,000× g) to remove dead cells and debris, followed by high-speed centrifugation (40,000–100,000× g) to pellet and enrich PDEVs (Shao et al., 2018). UC is particularly well-suited for large-volume processing, enabling the isolation of PDEVs from hundreds of milliliters of plant extract in a single run (Pinedo et al., 2021). It should be noted, however, that the inherent diversity of plant sources can lead to considerable variability in the size, concentration, and purity of UC-isolated PDEVs. Furthermore, critical parameters such as centrifugal force, duration, and temperature significantly influence vesicle composition and structural integrity (Rutter and Innes, 2020). Consequently, optimizing species-specific centrifugation conditions is essential to establish reproducible and efficient isolation protocols. Repeated centrifugation cycles may induce structural deformation or damage to PDEVs. The incorporation of a high-density isotonic cushion buffer at the bottom of the centrifuge tube can help mitigate such mechanical stresses and improve vesicle recovery (Li et al., 2017).

#### 3.2.2 Density gradient ultracentrifugation, DGC

Density gradient ultracentrifugation operates on the principle that particles denser than the medium sediment downward, while those with lower buoyancy float upward. A density gradient is established by layering solutions of progressively decreasing density from the bottom to the top of the centrifuge tube (Li et al., 2017). Commonly employed gradient media include sucrose, ioxaglate, polysucrose, and cesium chloride. Sucrose and iopamidol gradients separate particles largely based on size and shape, whereas cesium chloride gradients exploit intrinsic density variations among sample components (Bosslet et al., 1981). Iopamidol, a highly hydrophilic non-ionic compound known for its high density and low viscosity, is particularly suitable for continuous density gradient configurations, allowing for high-resolution and precise separations (Crescitelli et al., 2021). This technique offers notable advantages, including high separation efficiency and minimal vesicle deformation. However, the introduced density medium may co-purity with vesicles and interfere with downstream analytical procedures. Additionally, the method is generally not well-suited for processing large sample volumes.

#### 3.2.3 Ultrafiltration centrifugation, UFC

Ultrafiltration centrifugation (UFC) separates particles or polymers according to size and molecular weight through a membrane with defined pore sizes under hydrostatic pressure, allowing small molecules to pass while retaining larger species (Li et al., 2017). For instance, De Robertis et al. (2020) isolated and purified EVs from blueberries using a combination of 0.45 μm filtration and 1000 MWCO ultrafiltration. Compared to ultracentrifugation, ultrafiltration improves separation efficiency while better preserving the native morphology of PDEVs. This method also offers greater cost-effectiveness and commercial viability, as it does not require ultracentrifugation equipment. A notable limitation, however, is the tendency toward higher protein contamination in the final product (Yang D. et al., 2020). Ultrafiltration can be effectively integrated with other techniques to enhance sample purity. For example, Benedikter et al. (2017) demonstrated that combining ultrafiltration with size exclusion chromatography efficiently isolates EVs from cell culture media, yielding preparations suitable for downstream compositional and functional analyses.

#### 3.2.4 Size-exclusion chromatography, SEC

Size Exclusion Chromatography (SEC) is a chromatography technique that separates EVs according to their hydrodynamic size. The central component of SEC is a column packed with a porous stationary phase. This method functions based on molecular sieving, wherein molecules and particles smaller than the pore size enter the pores and are delayed, while larger entities such as EVs are excluded and elute earlier, thus achieving separation (Coumans et al., 2017). Guo et al. (2021) developed a streamlined two-stage SEC approach by optimizing CL-6B resin and a dual-elution strategy, which enabled highly efficient isolation of serum EVs with improved recovery and purity. The column demonstrated reusability for up to 10 cycles, offering a standardized and practical tool for clinical EV detection. Through time-based fractionation, SEC effectively separates PDEVs from contaminating proteins. This technique has been successfully applied to isolate PDEVs from multiple plant species, including cabbage and tomato (You et al., 2021; Bokka et al., 2020).

#### 3.2.5 Polymer-based precipitation

Polymer precipitation is a well-established and commercially viable technique for isolating small EVs under routine centrifugation conditions (Zeringer et al., 2015). This method employs hydrophilic polymers such as polyethylene glycol (PEG), which acts as a co-precipitant by reducing the solubility of EVs, leading to their aggregation and precipitation. Polymer precipitation offers several advantages, including straightforward scalability, high-throughput processing, and no requirement for specialized equipment, making it suitable for rapid EV isolation from a variety of biological sources (Bai et al., 2024). For instance, Kalarikkal (Kalarikkal et al., 2020) developed a PEG-6000-based protocol for purifying ginger-derived EVs that circumvents the need for costly ultracentrifugation steps. However, a major limitation of this approach is the generally low purity of the resulting isolates, as proteins, nucleic acids, and other non-vesicular contaminants often co-precipitate with EVs, considerably complicating downstream analyses (Ramirez et al., 2018).

#### 3.2.6 Immunoaffinity capture-based technique

The immunoaffinity capture method operates through the specific binding of exosomal surface markers to antibodies immobilized on a solid-phase matrix, enabling highly selective isolation (Chai et al., 2025). This technique can enrich exosome subpopulations expressing specific target antigens, resulting in isolates of high purity and specificity. For instance, Ostenfeld et al. (2016) successfully captured a nanoparticle subset derived from cancer cells using anti-epithelial cell adhesion molecule (EpCAM) antibodies as affinity ligands. Similarly, Kang et al. (2020) employed an immunoaffinity-based microfluidic platform to isolate and analyze circulating tumor cells and cancer exosomes from melanoma patient blood samples. In the context of plant research, He et al. (2021) used *Arabidopsis thaliana* as a model and purified TET8-positive EVs via immunomagnetic beads coated with TET8 antibodies, which revealed a distinct enrichment of sRNAs and RNA-binding proteins within the isolated vesicles. Immunoaffinity-based enrichment offers notable advantages such as rapid processing, operational simplicity, and high specificity. However, it is also associated with several limitations, including prolonged handling times, high costs, limited throughput, modest yields, and dependence on antibody availability. Particularly for PDEVs, the identity of specific marker proteins remains inadequately characterized, and commercial antibodies are scarce, further constraining the broad application of this method.

#### 3.2.7 Other separation methods

Field flow fractionation (FFF) represents an alternative isolation technique in which an external force field is applied perpendicularly to the direction of sample flow, enabling separation based on differences in particle size and molecular weight. In contrast to traditional SEC, FFF operates without a stationary phase, under low system pressure, and with minimal shear forces, thereby preserving sample integrity throughout the separation process. As the sample flows through a thin, flat channel, a size-dependent gradient forms perpendicular to the flow direction, causing components of different sizes to elute at distinct times and facilitating high-resolution separation (Sitar et al., 2015). Oeyen et al. (2018) introduced an advanced characterization approach combining asymmetric flow field-flow fractionation with ultraviolet and multi-angle light scattering detection (AF4/UV-MALS). Further investigations have demonstrated that AF4 technology—with its high separation resolution and minimal interference—holds significant promise for the precise isolation and subsequent functional analysis of PDEVs, both as a standalone method and in combination with other techniques.

Aqueous two-phase systems (ATPS) are formed by mixing two polymers or a polymer with a salt solution, resulting in the formation of immiscible aqueous phases. Savci et al. (2021) developed an ATPS-based approach to isolate grapefruit-derived PDEVs while effectively depleting both proteinaceous and non-proteinaceous contaminants. This method offers a gentle and scalable route for obtaining high-purity vesicles.

Recent studies suggest that integrating multiple isolation techniques can yield superior separation outcomes for EVs. Yang M. et al. (2020) successfully combined electrophoresis with dialysis bags to isolate lemon-derived EVs, achieving vesicles comparable in both size and yield to those obtained through conventional ultracentrifugation. In another approach, Woith and Melzig (2019) effectively removed soluble protein contaminants by coupling differential centrifugation with agarose gel electrophoresis, thereby significantly enhancing sample purity.

### 3.3 Storage of PDEV

Stability and storage represent critical factors influencing the reproducibility and scalability of EV research. Current storage approaches for EVs mainly include cryopreservation, lyophilization, and spray drying (Zhang et al., 2020). Lorincz et al. (2014) systematically assessed the stability of neutrophil-derived EVs under various storage conditions and reported that although short-term storage at −80 °C preserves the physical integrity of EVs, it does not fully maintain their functional activity. Key biological functions—such as antimicrobial capacity and cellular uptake efficiency—were observed to decline partially under these conditions. Moreover, repeated freeze–thaw cycles or the use of cryoprotectants like DMSO or glycerol may induce vesicle lysis or functional inactivation. Therefore, the use of freshly isolated EVs is highly recommended for functional assays. The storage stability of PDEVs is affected by multiple factors, including temperature, duration, preservatives, and freeze–thaw cycles. Kim K. et al. (2022) demonstrated that the addition of TMO preservative enabled leaf-derived EVs stored at 4 °C for 4 weeks to retain consistent particle size and minimize protein degradation. In a study by Leng et al. (2023), 4 °C was identified as optimal for short-term storage of blueberry PDEVs, whereas −80 °C was more suitable for long-term preservation.

## 4 Tissue engineering with PDEV

**FIGURE 4:**
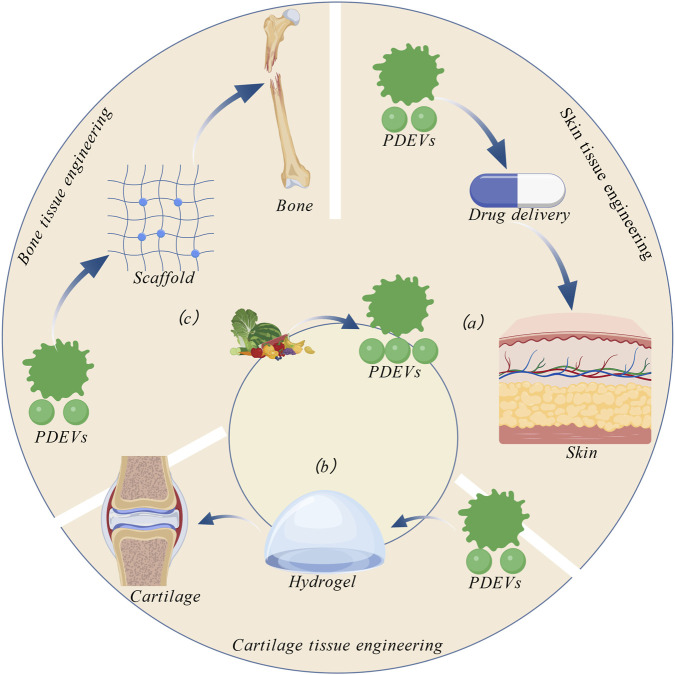
PDEVs and Tissue Engineering. PDEVs serve as natural bioactive carriers. Engineered PDEVs can exert positive regulatory and therapeutic effects on **(a)** skin, **(b)** cartilage, and **(c)** the skeletal system.

In the field of tissue engineering and regenerative medicine, PDEVs are gaining increasingly widespread application due to their exceptional biocompatibility, structural stability, functional diversity, and pivotal role in intercellular communication. To achieve effective retention and controlled release of PDEVs within local tissues, suitable delivery carriers are often required. Among these, hydrogels have emerged as an ideal vesicle carrier due to their unique three-dimensional network structure, highly tunable physicochemical properties, and excellent tissue-mimetic characteristics. Hydrogels can efficiently load and physically encapsulate PDEVs, protecting vesicle integrity while enabling sustained, localized release. Typically lacking significant physiological activity themselves, hydrogels primarily serve as carriers, delivery vehicles, and positioning agents. Building on this foundation, PDEVs have demonstrated remarkable regenerative efficacy in diverse tissue repair models. Their success in healing skin, bone, cartilage, nerve, and cardiovascular structures underscores their broad therapeutic potential (As shown in Figure 4). Crucially, the therapeutic efficacy of PDEVs is closely linked to their cargo of bioactive phytochemicals inherited from the parent plant. Depending on their botanical origin, PDEVs are rich in specific secondary metabolites such as phenolics, flavonoids, alkaloids, and terpenoids, which exhibit unique biological activities ranging from anti-inflammatory and antioxidant effects to bone induction (Zhang et al., 2016; Zeng et al., 2018). For instance, PDEVs derived from medicinal herbs often encapsulate high concentrations of the plant’s active compounds, thereby functioning as natural, high-efficiency delivery systems that enhance the bioavailability of these phytochemicals in tissue repair contexts. For example, in skin regeneration, PDEVs can be incorporated into topical formulations to accelerate wound healing through enhanced cell proliferation and angiogenesis. In cartilage repair, PDEV-loaded hydrogels promote chondrogenic differentiation, stimulate the synthesis of cartilage-specific extracellular matrix components, and confer anti-inflammatory and chondroprotective effects. In bone regeneration, the incorporation of PDEVs into bioactive scaffolds enhances the osteogenic differentiation of stem cells, stimulates mineralization and bone matrix deposition, while their immunomodulatory properties help establish a favorable osteogenic microenvironment.

### 4.1 Skin tissue engineering

**FIGURE 5:**
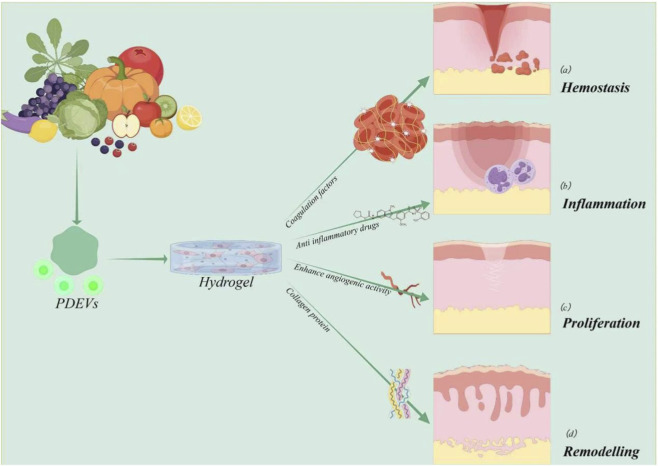
PDEVs exert multifaceted effects in wound healing PDEVs simultaneously influence multiple stages of wound healing— **(a)** PDEVs primarily promote coagulation by interacting with blood cells, coagulation factors, and platelets; **(b)** PDEVs create a healing-favorable microenvironment through anti-inflammatory, antimicrobial, antioxidant, and immunomodulatory properties, while also delivering anti-inflammatory drugs to reduce wound inflammation; **(c)** PDEVs promote angiogenesis, enhance wound blood supply, and accelerate healing; **(d)** PDEVs facilitate complete wound closure with scar formation by upregulating collagen type I and remodeling collagen type III.

The skin, being the largest organ of the human body, serves as the primary interface with the external environment and plays essential roles in providing a critical barrier against environmental insults and maintaining internal homeostasis. However, extensive skin damage, such as that seen in diabetic pathologies which frequently result in chronic wounds, can severely disrupt the normal healing process, driving research in skin tissue engineering to focus on strategies that accelerate repair and restore function. The process of cutaneous wound healing unfolds through four dynamic and overlapping phases: hemostasis, inflammation, proliferation, and remodeling. Each of these stages involves tightly regulated biological events that collectively facilitate the complete restoration of skin integrity and barrier function (As shown in Figure 5). Notably, EVs derived from various plant sources have shown considerable therapeutic potential in animal models, particularly in the treatment of diabetic wounds and full-thickness skin defects. PDEVs demonstrate multi-target regulatory capabilities in skin repair, simultaneously influencing hemostasis, anti-inflammation, angiogenesis, and remodeling phases—an advantage synthetic drugs struggle to match.

During the hemostatic phase of wound healing, vasoconstriction and coagulation cascades are initiated, leading to clot formation. PDEVs contribute to procoagulant activity primarily through interactions with blood cells, coagulation factors, and platelets. Ding et al. (2025) developed an *in situ* sprayable hydrogel (CC-TFP) by integrating carbon quantum dots (C-dots) derived from Sophora flower charcoal—a traditional hemostatic herb—and EVs isolated from cactus (C-EVs), known for their detoxifying and anti-edema properties. The hydrogel undergoes rapid cross-linking via tannic acid (TA), Fe^3+^, and acrylamide (AM), forming a supramolecular dual-network structure with strong tissue adhesion, autonomous self-healing capacity, and on-demand removability. This composite material promotes fibrin generation by activating endogenous coagulation pathways through C-dots, while the TA–Fe^3+^ complex facilitates physical occlusion via phenolic hydroxyl-mediated adhesion and cross-linking with tissue and blood components. Its porous architecture further supports platelet enrichment and aggregation, resulting in synergistic hemostatic efficacy. In a mouse tail-amputation model, the hydrogel achieved rapid hemostasis within 40 s, demonstrating advantages such as ease of application via spraying and immediate therapeutic action.

The inflammatory phase is a necessary stage in wound healing; however, excessive or prolonged inflammation can impede recovery and become detrimental. The accumulation of ROS disrupts macrophage polarization and delays the transition from the inflammatory to the proliferative phase. Bacterial infections further complicate healing by forming biofilms and impairing angiogenesis, leading to persistent inflammation, tissue necrosis, and increased therapeutic challenges (Peng et al., 2021). PDEVs exhibit anti-inflammatory, antibacterial, antioxidant, and immunomodulatory properties that can ameliorate excessive inflammatory responses and foster a microenvironment conducive to healing. The immunomodulatory effects of PDEVs are primarily achieved by regulating key signaling pathways. For instance, lemon-derived PDEVs alleviate inflammation by inhibiting the ERK/NF-κB pathway, thereby reducing TNF-α and IL-6 levels (Raimondo et al., 2022). Similarly, ginseng-derived PDEVs (GENs) suppress NF-κB activation and promote a shift toward an anti-inflammatory immune phenotype (Kim et al., 2023b). Studies have shown that PDEVs from botanical sources such as lemon, cabbage, aloe vera, pomegranate, kudzu root, and turmeric possess significant anti-inflammatory activities (Sanchez-Lopez et al., 2022; You et al., 2021; Raimondo et al., 2022; Ramirez et al., 2024; Wu et al., 2022; Gao C. et al., 2022). Specifically, ginger-derived PDEVs (GDNVs) have been shown to contain high levels of 6-gingerol and 6-shogaol, bioactive phenols known to effectively mitigate oxidative stress and inflammation, thereby accelerating the healing of inflammatory wounds (Bischoff-Kont and Fürst, 2021; Hong et al., 2020). Similarly, grapefruit-derived PDEVs are enriched with naringin and naringenin, flavonoids that not only exert anti-inflammatory effects but also play a pivotal role in promoting angiogenesis during the wound healing process (Wang et al., 2014; Zeng et al., 2018). For instance, Wei et al. (2025a) encapsulated wintergreen-derived PDEVs (ENs) loaded with the homologous drug oregonin I (ORI) into a semi-interpenetrating network hydrogel (ORI/ENs/Gel). This formulation targeted NLRP3 inflammasome inhibition and exerted synergistic antibacterial and anti-inflammatory effects, markedly accelerating the healing of radiation-induced oral mucositis. Additionally, Miya et al. (2025) incorporated nano-vesicles from aloe, ginger, and neem fruit (OXY-ExoAloe) into a chitosan–polyvinyl alcohol (PVA) nano-membrane (OXY-NMAloe) to enhance oxygen delivery to hypoxic wounds, such as diabetic foot ulcers. This membrane facilitates targeted delivery of therapeutic nanovesicles, improves cellular metabolism, attenuates oxidative stress, and promotes tissue regeneration through paracrine mechanisms. As an efficient delivery vehicle, it not only transports bioactive compounds but also establishes an optimal wound microenvironment for enhanced healing.

The proliferative phase of wound healing occurs concurrently with and overlaps the inflammatory phase. During this stage, key processes such as re-epithelialization and angiogenesis take place, culminating in the formation of granulation tissue. PDEVs facilitate this phase by promoting cellular proliferation, migration, and differentiation, thereby supporting neotissue formation and repair. Specific biomolecular components within PDEVs have been shown to enhance angiogenic activity, improve wound perfusion, and accelerate overall healing. For instance, Zhao et al. (2025) incorporated Morinda Officinalis-derived PDEVs (MoEVLPs) into a hydrogel system to extend their local retention and activity. They demonstrated that the MoEVLP-loaded hydrogel promotes angiogenesis and accelerates wound healing through MAPK-MEK1/2-mediated activation of YAP1 and HIF-1α signaling pathways. Studies have demonstrated that PDEVs isolated from grapefruit, aloe vera, soapberry, wheat, and ginseng enhance the tubulogenic capacity of human umbilical vein endothelial cells (HUVECs) and stimulate angiogenesis, underscoring their therapeutic potential in wound management (Savci et al., 2021; Kim and Park, 2022; Sahin et al., 2019; Yang et al., 2023). Notably, the hydrogel formulation markedly amplified pro-angiogenic effects compared to free MoEVLPs, highlighting its promising applicability in chronic wound treatment. Research by Di Raimo et al. (2024) confirms that a mixture of PDEVs, isolated from five organic fruits including grapes, blood oranges, tangerines, papayas, and pomegranates, is rich in key antioxidants such as superoxide dismutase, catalase, glutathione, ascorbic acid, and citric acid. These PDEVs are effectively internalized by human skin fibroblasts. Upon cellular uptake, they restore mitochondrial membrane potential under oxidative stress, reduce superoxide anion levels, and upregulate Sirtuin-1 expression. These actions collectively promote cell migration, enhance collagen I production, and stimulate matrix metalloproteinase-9 generation, thereby accelerating wound healing and highlighting the mixture’s notable potential in antioxidant defense, anti-aging, and skin repair. Research conducted by You et al. (2021) demonstrated that PDEVs derived from Chinese cabbage (Cabex) and red cabbage (Rabex) markedly enhance the proliferation of human dermal fibroblasts (HDF) and human keratinocytes (HaCaT). Furthermore, these vesicles effectively suppress STS-induced apoptosis and reduce caspase-3 activity. These findings indicate their potential in promoting skin regeneration and modulating anti-apoptotic pathways.

During the final stage of wound healing, known as the remodeling phase, scar formation takes place as type III collagen is progressively remodeled into type I collagen, and wound closure is completed. The balanced activity of fibroblasts, driven by PDEVs, is crucial in this phase. Studies have shown that PDEVs derived from Peruvian ground cherry fruit enhance the proliferation and migration of human dermal fibroblasts, upregulate type I collagen expression, and exhibit considerable potential for improving chronic wound healing (Natania et al., 2025). In their research, Wang T. et al. (2025) isolated and characterized coriander-derived PDEVs (CDENs) and engineered a CDEN-based hydrogel with sustained-release capacity and excellent biocompatibility. Throughout the remodeling phase, this hydrogel significantly increased the density and organization of collagen deposition, and promoted the regeneration of skin appendages including hair follicles and blood vessels, and facilitated the transition of fibroblasts to a regeneration-associated phenotype, ultimately supporting functional skin reconstruction.

PDEVs exert multi-target regulatory effects across all four phases of wound healing—hemostasis, inflammation, proliferation, and remodeling. Engineering strategies such as hydrogel-based encapsulation promote sustained and prolonged release of PDEVs at wound sites, thereby enhancing their therapeutic efficacy. The 3D moist environment they provide synergistically creates an optimal healing microenvironment with PDEVs, addressing the core challenge of chronic wound non-healing. This approach also opens new horizons for skin tissue engineering applications (Huan et al., 2021).

### 4.2 Cartilage tissue engineering

Cartilage is a specialized, load-bearing connective tissue that provides essential cushioning and lubrication during joint movement. Unlike cutaneous tissue, however, cartilage is avascular, Alymphatic, and aneural, which severely restricts its innate regenerative capacity. Extensive cartilage defects caused by conditions such as osteoarthritis, trauma, or congenital abnormalities often lack the capacity for spontaneous healing. Consequently, these defects frequently progress to chronic pain and functional disability. In recent years, PDEVs, also referred to as plant exosome-like nanovesicles, have emerged as a novel class of bioactive nanomaterials with promising potential for cartilage regeneration. Studies indicate that PDEV-based tissue engineering strategies, whether through functionalized scaffolds, hydrogels, or cell-based therapies, elicit favorable therapeutic outcomes in both *in vitro* chondrocyte cultures and *in vivo* animal models of cartilage injury.

PDEVs facilitate the growth and maturation of neocartilage by delivering bioactive molecules, such as growth factors, thereby establishing a regenerative microenvironment conducive to chondrogenesis. Accumulating evidence supports the potential of PDEVs in promoting cartilage tissue formation. For instance, Yildirim et al. (2024) isolated EVs from lemons and tomatoes and experimentally identified 100 μg/mL as the optimal concentration for enhancing cell proliferation. Specifically, tomato-derived PDEVs (TELVs) not only promoted the chondrogenic differentiation of adipose-derived stem cells but also upregulated the expression of key chondrogenesis-related genes, including COL2, ACAN, and SOX9. In contrast, lemon-derived exosome-like vesicles (LELVs) suppressed the expression of genes associated with chondrogenic differentiation. These findings highlight the significant and source-dependent regulatory role of PDEVs in the chondrogenesis of adipose-derived stem cells. Based on these results, PDEVs represent a promising candidate for clinical applications in cartilage regeneration and may offer novel therapeutic strategies for the treatment of osteoarthritis (OA).

PDEVs also exhibit significant anti-inflammatory properties. Inflammation plays a central role in the pathogenesis of OA (Ansari et al., 2020; Liu et al., 2022). Several studies have demonstrated that certain PDEVs possess notable anti-inflammatory activities. For example, Kim et al. (2023b) reported that ginseng-derived PDEVs (GENs) reduce the production of inflammatory mediators such as TNF-α and IL-6 under inflammatory conditions by suppressing NF-κB pathway activation and modulating the immune microenvironment toward an anti-inflammatory phenotype. In another study, Wei et al. (2023) identified that turmeric-derived PDEVs encapsulate curcumin, a hydrophobic polyphenol with potent anti-inflammatory properties. These vesicles serve as a natural vehicle to increase curcumin’s solubility and stability. Delivered via PDEVs, curcumin efficiently inhibits NF-κB signaling via activation of the Nrf2/HO-1 axis, downregulates pro-inflammatory factors such as COX-2 and MMPs, and thereby mitigates OA-related inflammation and symptoms (Wang and He, 2022; Henrotin et al., 2014).

The progressive degeneration and erosion of articular cartilage represent hallmark pathological features of osteoarthritis (OA) (Rim et al., 2020). Emerging evidence indicates that miRNAs encapsulated in PDEVs regulate the plasticity of surviving cells in injured regions by modulating the extracellular matrix (ECM), thereby promoting chondrocyte differentiation, proliferation, and ultimately facilitating cartilage regeneration (Sen and Ghatak, 2015). Yildirim et al. (2024) demonstrated that tomato-derived EVs enhance the expression of key chondrogenic markers—including aggrecan (ACAN), SRY-box transcription factor 9 (SOX9), and cartilage oligomeric matrix protein (COMP)—thereby supporting cartilage repair. Their findings further suggest that PDEVs can deliver growth factors to chondrocytes, helping to establish a chondroinductive microenvironment conducive to the growth and maturation of neocartilage. In a related study, Chen et al. (2022) encapsulated spinach-derived nanocrystalline units (NTU) within chondrocyte membranes (CM) and introduced them into chondrocytes. They observed that CM-NTU significantly increased intracellular ATP and NADPH levels under natural light illumination, enhancing anabolic metabolism in degenerated chondrocytes and thereby improving cartilage homeostasis while attenuating OA progression. Additionally, PDEVs derived from antler stem cells (ASC-Exos) have been shown to mediate factors such as TF, S100A4, and IGF-1, promoting the proliferation and migration of chondrocytes and bone marrow-derived mesenchymal stem cells (BMSCs) while maintaining chondrocyte phenotype. In an *in vivo* study, Zhou et al. (2024) implanted ASC-Exos-loaded hydrogels into cartilage defects in rats and confirmed their robust efficacy in promoting cartilage regeneration.

**FIGURE 6:**
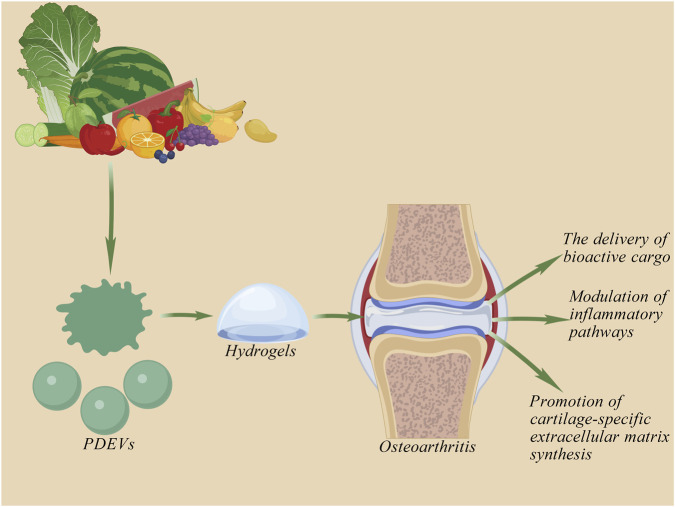
Repair Mechanism of PDEV on the Cartilage System. PDEV promotes cartilage repair by delivering growth factors, modulating inflammatory responses, and stimulating the synthesis of cartilage-specific extracellular matrix.

PDEVs facilitate cartilage repair through multiple mechanisms, including the delivery of bioactive cargo such as growth factors, modulation of inflammatory pathways, and promotion of cartilage-specific extracellular matrix synthesis (As shown in Figure 6).

### 4.3 Bone tissue engineering

**FIGURE 7:**
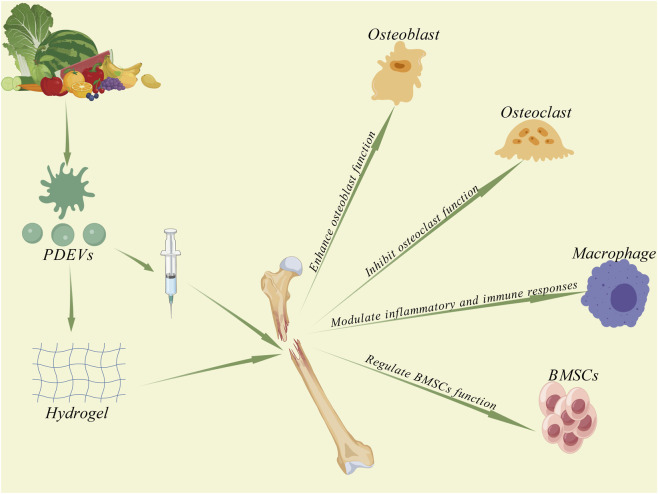
Mechanism of PDEV in Fracture Healing. PDEV intervenes in fracture healing by enhancing osteoblast differentiation and mineralization, suppressing osteoclast differentiation and activity, modulating inflammatory and immune responses, and regulating the function of BMSCs.

PDEVs offer a distinct strategic approach to bone repair by concurrently targeting two critical processes: promoting osteogenic differentiation and suppressing osteoclastic activity. This bidirectional regulation, combined with their ability to foster a supportive immune microenvironment, underlies their exceptional efficacy in facilitating bone regeneration. In the field of bone regeneration, most bone injuries can be managed using standard clinical interventions such as reduction, internal and external fixation, and rehabilitation therapy. However, the repair of large bone defects and complex fractures remains a considerable challenge, particularly in patients with systemic comorbidities such as osteoporosis, diabetes, or malignancies. Current bone graft materials, primarily autologous and allogeneic grafts, are associated with significant limitations; these include donor site morbidity, limited supply, insufficient osteoinductivity, and potential infection risks. Moreover, existing regenerative strategies often exhibit limited efficacy, which can lead to serious complications including delayed union or nonunion. Currently available pharmacological options for enhancing fracture healing are also scarce, underscoring the urgent need to develop novel and effective therapeutic strategies to promote bone regeneration. Recent studies suggest that PDEVs hold promising potential in this context by enhancing osteogenic differentiation and mineralization, inhibiting osteoclastogenesis, modulating immune responses, and stimulating the proliferation of BMSCs through the regulation of specific signaling pathways such as BMP/Smad, PI3K/Akt, and MAPK (As shown in Figure 7).

PDEVs enhance osteogenic differentiation and mineralization through multiple bioactive mechanisms. Xu et al. (2025) isolated goji berry-derived PDEVs (GqDNVs) using sucrose density gradient centrifugation and demonstrated that GqDNVs promote MC3T3-E1 cell proliferation, increase alkaline phosphatase (ALP) activity, upregulate osteogenic markers including osteopontin (OPN) and bone gla protein (BGP), and downregulate sclerostin—a negative regulator of bone formation. *In vivo* studies further revealed that GqDNVs facilitate fracture healing in mice by modulating the bone regenerative microenvironment via the PI3K/Akt/mTOR/p70S6K/4EBP1 pathway, exerting dual anti-inflammatory and osteogenic effects. In another study, Hwang et al. (2023) showed that yam-derived PDEVs (YNVs) significantly enhance osteogenic differentiation and mineralization through activation of the BMP-2/p38 MAPK/Runx2 signaling pathway, outperforming conventional osteoinductive agents such as diosgenin. Using DiR fluorescent tracing, they confirmed that orally administered YNVs are absorbed via the intestinal tract and specifically accumulate in bone tissue. Their osteogenic effects were attributed to endogenous vesicular mRNA and proteins rather than saponin constituents, providing novel mechanistic insight into plant vesicle-mediated bone repair. Additionally, Zhao et al. (2024) highlighted the role of specific flavonoids in bone repair, reporting that PDEVs derived from Drynaria roosii (RDNVs) are enriched with naringenin. This phytochemical specifically targets and activates the estrogen receptor alpha (ERα) pathway, promoting the proliferation and osteogenic differentiation of human BMSCs. Treatment with RDNVs effectively ameliorated osteoporosis in an ovariectomized (OVX) mouse model. Wei et al. (2025b) synthesized naringenin-copper carbon dots (Nar-CuCDs) via a hydrothermal method and incorporated them into a dual-crosslinked hydrogel scaffold. This composite system scavenges ROS, induces M2 macrophage polarization to improve the osteoimmune microenvironment, exhibits antibacterial properties, and synergistically enhances osteogenic differentiation of rat BMSCs, significantly improving bone defect repair both *in vitro* and *in vivo*. Furthermore, Zhao et al. (2021) developed naringenin-loaded silk fibroin/hydroxyapatite composite scaffolds (NG/SF/HAp) using salt-leaching technology. These scaffolds facilitated osteogenic differentiation and angiogenesis of human umbilical cord mesenchymal stem cells (hUCMSCs), markedly enhancing bone regeneration *in vivo* and *in vitro*, while demonstrating excellent biocompatibility and controlled degradability.

PDEVs exhibit inhibitory effects on osteoclast differentiation. Seo et al. (2023) isolated ginseng-derived PDEVs (GDNs) via sucrose gradient centrifugation and demonstrated that GDNs significantly suppress the formation of TRAP^+^ osteoclasts by inhibiting RANKL-induced phosphorylation of JNK, ERK, and IκBα, as well as downregulating the expression of key osteoclastogenic transcription factors NFATc1 and c-Fos. *In vivo* studies further revealed that GDNs effectively attenuate bone loss and improve bone microarchitecture in a mouse model of osteoporosis. Notably, the osteoprotective effect of GDNs is largely attributed to their cargo of ginsenosides, a class of steroid glycosides and triterpene saponins. Specifically, GDNs enriched with ginsenoside Rb1 showed significantly stronger inhibition of osteoclast differentiation compared to the monomeric saponin alone, indicating that vesicular encapsulation enhances the bioavailability and bioactive efficacy of these phytochemicals. As nanoscale carriers, GDNs can be co-delivered with scaffolds or hydrogels, offering a novel plant-based targeted strategy for the treatment of osteoporosis and bone tissue engineering. In a complementary study, Park et al. (2023) found that plum-derived PDEVs promote osteoblast differentiation while simultaneously inhibiting osteoclast activation through the BMP-2/p38/JNK/Smad1–Runx2 signaling pathway. These findings highlight the potential of plant-derived vesicles as bidirectional regulators in bone remodeling and engineering.

PDEVs play a unique role in bone repair through an exceptional capacity for bidirectional and synergistic regulation. Research by Park et al. (2023) demonstrated that PDEVs directly promote osteoblast differentiation, mineralization, and bone matrix formation by activating the BMP-2/Smad/MAPK signaling pathway, which upregulates the key transcription factors Runx2 and Osterix. Conversely, PDEVs inhibit core osteoclastogenic factors, including PPAR-γ and NFATc1, thereby reducing the activity of bone-destroying osteoclasts. These dual actions are synergistic: promoting osteogenesis establishes a healthy bone microenvironment, while suppressing osteoclasts prevents matrix degradation. Together, they disrupt the vicious cycle of bone loss and shift the bone remodeling balance toward formation, resulting in a net bone gain. This mechanism offers a novel and promising strategy for treating bone-related diseases such as fractures and osteoporosis.

PDEVs play a pivotal role in modulating immune and inflammatory responses, which are critical processes in fracture healing and bone defect repair (Wildemann et al., 2021). The immunomodulatory capacity of PDEVs offers promising applications in bone tissue engineering. For instance, Vanessa et al. (2024) demonstrated that PDEVs isolated from bayberry effectively reduce the production of pro-inflammatory M1 macrophage markers and promote polarization toward the anti-inflammatory M2 phenotype, thereby mitigating chronic inflammation *in vivo*. In a complementary study, You et al. (2021) reported that PDEVs derived from Cabex and Rabex not only enhance mammalian cell proliferation but also suppress inflammatory responses in immune cells and inhibit apoptosis in human keratinocytes and fibroblasts. Importantly, these vesicles successfully delivered encapsulated therapeutic agents into human cells, underscoring their potential as efficient drug delivery vehicles. Together, these findings provide compelling evidence for the broad applicability of PDEVs as innovative therapeutic biomaterials in regenerative medicine (You et al., 2021).

PDEVs play a regulatory role in the function of BMSCs, which act as central cellular and signaling mediators in fracture healing. BMSCs orchestrate bone repair through three fundamental mechanisms: supplying osteoblasts, exerting immunomodulatory effects, and secreting growth factors to promote angiogenesis. Their functional status and activity are critical determinants of the rate and quality of bone regeneration (Xu et al., 2023). Gupta et al. demonstrated that PDEVs derived from Cissus quadrangularis (CQ-ELNs) significantly enhance osteogenic differentiation in human mesenchymal stem cells (hMSCs) and myoblasts (C2C12), as evidenced by increased alkaline phosphatase (ALP) activity and enhanced calcium nodule formation. Additionally, CQ-ELNs promoted cellular migration, highlighting their potential as a novel plant-based therapeutic platform for bone tissue engineering. In a complementary study, Perut et al. (2021) reported that strawberry-derived PDEVs, enriched with vitamin C, mitigate oxidative stress and stimulate the proliferation of BMSCs. These findings further underscore the capacity of PDEVs to modulate stem cell behavior and support regenerative processes.

PDEVs exhibit considerable promise in bone tissue engineering owing to their nanoscale structure, high biocompatibility, and efficient biomolecule delivery capabilities. Their multifunctional roles include: (1) promoting osteogenic differentiation and mineralization; (2) inhibiting osteoclast formation and activity; (3) modulating inflammatory and immune responses; and (4) regulating the behavior and function of BMSCs. Notably, PDEVs possess unique therapeutic advantages in bone repair, stemming from the synergistic effects of their bidirectional regulation and immunomodulatory functions. They simultaneously promote osteogenic differentiation and suppress osteoclast activity. Furthermore, by modulating the immune microenvironment, they establish a favorable repair environment, thereby achieving efficient bone regeneration. Additionally, PDEVs combine therapeutic and delivery functions into a single platform, offering exceptional stability and favorable safety profiles. These attributes position PDEVs as promising candidates to address certain technical challenges associated with current bone regeneration strategies.

### 4.4 Other

The therapeutic potential of PDEVs in neurological disorders is attracting growing interest, largely due to their innate capacity to cross the blood–brain barrier and deliver bioactive molecules directly to the central nervous system. For example, ginseng-derived and grape-derived exosome-like nanoparticles have been shown to efficiently traverse the blood–brain barrier, delivering ginsenosides or the chemotherapeutic agent doxorubicin into the brain to suppress glioma progression. Engineered PDEVs further enhance targeted drug delivery, effectively mitigating oxidative stress and inflammatory responses (Kim et al., 2023a; Niu et al., 2021). In a Parkinson’s disease model, carrot-derived PDEVs (CDEs) exhibited antioxidant activity and suppressed caspase-3-dependent apoptotic pathways (Kim and Rhee, 2021). Similarly, Lycium barbarum-derived PDEVs loaded with isoliquiritin promoted M2 macrophage polarization and neural regeneration via activation of the pAKT/AKT pathway, leading to functional recovery in spinal cord injury models (You et al., 2021). Additionally, oat-derived PDEVs (OATNs) have been found to alleviate alcohol-induced neuroinflammation and memory deficits by inhibiting the NF-κB/Syk pathway through β-glucan binding to hippocampal CD11b (HPCA) (Xu et al., 2022).

Cardiovascular disease remains a leading cause of mortality worldwide (Ran et al., 2025). PDEVs have shown promising cardioprotective effects by attenuating cardiomyocyte apoptosis through the reduction of ROS production and activation of antioxidant pathways such as Nrf2. PDEVs isolated from carrots, blueberries, and bitter melon have all demonstrated such protective properties (Kim and Rhee, 2021; Ye et al., 2024; Zhao et al., 2022). For instance, Zhou et al. (2025) developed a goji berry-derived nanovesicle-fibrin gel (GqDNVs-gel) that significantly improved survival rates after myocardial infarction, enhanced cardiac function, and regulated lipid metabolism via suppression of the p38 MAPK/NF-κB p65 pathway. Beyond cardiomyocyte protection, PDEVs also modulate vascular smooth muscle cell (VSMC) phenotypic switching by inhibiting aberrant proliferation and migration, thereby delaying the progression of atherosclerosis and restenosis. Tomato-derived PDEVs, for example, have been shown to regulate VSMC function through the Keap1/Nrf2 and Mef2D signaling pathways (Shen et al., 2025). In a study by Liu et al. (2024), green tea-derived PDEVs encapsulating antagomir-HAAPIR were used to deliver piRNA stably to aortic lesion sites. This strategy effectively suppressed VSMC phenotypic switching via the Mef2D pathway, leading to a marked reduction in the incidence of aortic dissection and improved survival rates.

PDEVs have emerged as a versatile therapeutic platform in regenerative medicine due to their innate nanoscale structure, low immunogenicity, and ability to cross physiological barriers like the blood-brain barrier. Compared to synthetic carriers, PDEVs demonstrate superior cellular uptake and biocompatibility, capable of efficiently delivering diverse cargoes including nucleic acids, proteins, and small molecule drugs. In tissue engineering applications, PDEVs are frequently incorporated into hydrogels or bioactive scaffolds to achieve sustained, localized release. This composite strategy has proven effective in promoting skin wound healing, cartilage repair, and bone regeneration by maintaining vesicle integrity within the local microenvironment. Additionally, PDEVs serve as potent vehicles for vaccine antigens, such as HBsAg, significantly enhancing immunogenicity (Kim et al., 2025). These findings collectively underscore the translational potential of PDEVs as a novel and promising drug delivery strategy in tissue engineering and regenerative medicine.

In the fields of tissue engineering and targeted therapy, engineered biomaterials and membrane fusion technologies provide innovative strategies for advanced drug delivery systems. Three-dimensionally bioprinted scaffolds allow localized drug loading and sustained release of therapeutic agents. For example, gelatin methacryloyl (GelMA) scaffolds loaded with lemon-derived PDEVs (CLEVs), combined with systemic chemotherapy, synergistically inhibited tumor viability, aggregation, and migration in a triple-negative breast cancer model, while also promoting fibroblast proliferation to facilitate post-surgical tissue regeneration (Cui et al., 2024). To further enhance the targeting specificity and therapeutic efficacy of PDEVs, membrane fusion technology has emerged as a key engineering strategy. Techniques such as extrusion, freeze-thaw cycles, or ultrasonication enable the fusion of PDEVs with membranes from other cell types—including neutrophils and cancer cells—or with synthetic liposomes. The resulting hybrid vesicles retain the safety profile of plant-derived membranes while acquiring functional proteins from mammalian membranes, thereby conferring enhanced immune evasion and targeted homing capabilities (Sun et al., 2023). For instance, Ma et al. (2024) fused neutrophil membranes with ginseng-derived PDEVs to achieve targeted delivery of miRNA-182-5p, effectively mitigating sepsis-induced acute lung injury. Furthermore, surface modification with lipids or peptide ligands offers another promising avenue to improve the delivery efficiency of PDEVs. Together, these approaches provide essential platforms for the development of multifunctional biomimetic nanosystems, significantly broadening the prospects for precision drug delivery in regenerative medicine and targeted therapy. For a detailed comparison of PDEV separation methods and therapeutic applications, please refer to Table 2.

**TABLE 2:** Summary of PDEV isolation methods and therapeutic applications.

Plant source	Target tissue/Disease	Isolation method	Key therapeutic mechanism/Cargo	Research status	Ref.
Ginseng	Bone (Osteoporosis)	Sucrose gradient UC	Inhibits osteoclasts via RANKL/JNK pathway; Enriched in Ginsenosides	Preclinical (*In vivo*)	Seo et al. (2023)
Goji Berry	Bone (Fracture)	Sucrose gradient UC	Promotes osteogenesis via PI3K/Akt/mTOR pathway; Lowers sclerostin	Preclinical (Mice)	Sen and Ghatak (2015)
Yam	Bone (Osteoporosis)	Differential centrifugation	Enhances mineralization via BMP-2/p38 MAPK pathway	Preclinical (*In vivo*)	Chen et al. (2022)
Dryopteris	Bone (Osteoporosis)	Ultracentrifugation	Targets Erα to promote BMSC osteogenic differentiation	Preclinical (Mice)	Zhou et al. (2024)
Coriander	Skin (Wound Healing)	Differential centrifugation	Hydrogel-loaded; Promotes collagen deposition and appendage regeneration	Preclinical (*In vivo*)	Yang et al. (2023)
Grapefruit	Skin (Wound)/Delivery	Differential centrifugation	Functionalized lipids; Promotes angiogenesis; Low toxicity delivery	Preclinical	Zhang et al. (2025), He et al. (2021)
Aloe/Ginger	Skin (Diabetic Wound)	Polymer-based precipitation	Incorporated into nanofilms; Antioxidant & Anti-inflammatory	Preclinical (Rats)	Miya et al. (2025)
Tomato	Cartilage	Differential centrifugation	Upregulates chondrogenic genes (COL2, ACAN, SOX9)	Preclinical (*In vitro*)	Natania et al. (2025)
Turmeric	Cartilage (OA)	Differential centrifugation	Inhibits NF-kB via Nrf2/HO-1 axis; Anti-inflammatory	Preclinical	Yildirim et al. (2024)
Carrot	Cardiovascular (MI)	Ultracentrifugation	Antioxidant activity; Inhibits cardiomyocyte apoptosis	Preclinical (*In vitro*)	Vanessa et al. (2024)

## 5 Summary and outlook

PDEVs, as naturally sourced bioactive nanoscale delivery platforms, exhibit considerable promise in biomedical applications owing to their low immunogenicity, favorable biocompatibility, intrinsic antioxidant and anti-inflammatory activities, as well as their capacity for efficient drug loading and targeted delivery. However, the clinical translation of PDEV-based therapies still confronts several challenges. These include the lack of standardized isolation protocols, inherent heterogeneity in vesicle populations, immature large-scale production techniques, insufficient long-term storage stability, and the need for enhanced targeting specificity and delivery efficiency.

In the field of tissue engineering, PDEVs offer substantial advantages: they not only actively modulate the tissue microenvironment to facilitate the repair and regeneration of diverse tissues—including skin, bone, cartilage, and nerves—but also function as natural nanocarriers for the targeted delivery of therapeutic molecules, thereby integrating both regenerative and drug delivery functionalities. Moreover, their source materials are abundantly available and cost-effective, rendering them promising candidates for large-scale production. Nevertheless, several challenges remain to be addressed, such as the unclear mechanisms underlying tissue-specific targeting, insufficient systematic understanding of PDEV-host cell interactions (particularly regarding cross-species immunoreactivity mediated by plant glycans), a lack of comprehensive long-term *in vivo* safety assessments, and the absence of standardized manufacturing and quality control systems that meet clinical requirements.

Looking ahead, achieving sustainable and controllable production of plant raw materials is a promising strategy to enhance the quality and safety of PDEVs. Plant cell culture technology offers a viable pathway toward this goal. By providing standardized, axenic plant material under controlled conditions, this approach minimizes batch-to-batch variability and improves the reproducibility of PDEV isolation. Furthermore, such advances in plant bioengineering could enable the creation of tailored plant sources for specific therapeutic uses, thereby promoting the green and scalable production of PDEVs.

Overcoming these challenges is essential to advance PDEV based therapies and develop innovative delivery nanoplatforms. Engineering strategies commonly applied to mammalian EVs show potential for improving the targeting ability of PDEVs as drug carriers. For instance, functional ligands such as lipids or peptides can be used to modify PDEVs and enhance their targeting efficiency. Furthermore, membrane fusion techniques enable co-encapsulation of PDEVs with synthetic liposomes, mammalian EVs, or bacterial EVs to form hybrid vesicles with enhanced biological functions. Such precision engineering modifications hold promise for improving targeting specificity and enhancing immune evasion capabilities. While most current studies are limited to small animal models, robust preclinical validation in large animal models is a critical next step toward clinical translation. These models more closely replicate human physiology and pathology, allowing for evaluation of the safety and efficacy of PDEV based scaffold or hydrogel systems in repairing large bone defects, full thickness skin wounds, or articular cartilage injuries. Such studies will generate essential data on dosage, pharmacokinetics, and safety for future human trials. Given the heterogeneity resulting from different plant sources and isolation techniques, rigorous analysis and quality control of raw materials are necessary. Developing standardized isolation protocols will help yield uniform, stable, and high quality PDEVs. Establishing field wide consensus on key parameters for isolation, characterization, and storage, along with strict quality control standards, is crucial to ensure batch to batch consistency and product reliability.

In summary, although current research on PDEVs is still in its early exploratory stages, existing preliminary findings already underscore their promising therapeutic potential in tissue engineering. These insights provide a compelling foundation for the future development of more effective and safer regenerative medicine strategies. Through interdisciplinary collaboration focused on overcoming these critical challenges, PDEVs hold promise to evolve from a promising experimental tool into a transformative class of clinical therapeutics in the near future, ultimately advancing the field of regenerative medicine.

## Glossary

4EBP1: Eukaryotic Translation Initiation Factor 4E-Binding Protein 1
ADNVs: Aloe-derived PDEVs
AF4: Asymmetric Flow Field-Flow Fractionation
AFM: Atomic Force Microscopy
ALP: Alkaline Phosphatase
AM: Acrylamide
ASC-Exos: Antler Stem Cell-derived PDEVs
ATP: Adenosine Triphosphate
ATPS: Aqueous Two-Phase System
BGP/OCN: Bone Gla Protein/Osteocalcin
BMP: Bone Morphogenetic Protein
BMSCs: Bone Marrow-derived Mesenchymal Stem Cells
cAW: centrifugation-based washing
Cabex: Cabbage-derived PDEVs
C-CP: Capillary channel polymer
C-dots: Carbon dots
CDEs: Carrot-derived PDEVs
CDENs: Coriander-derived PDEVs
CELNs: Celery-derived PDEVs
C-EVs: Cactus Extracellular Vesicles
CLEVs: Lemon-derived PDEVs
CM: Chondrocyte Membrane
COL2/ACAN: Collagen Type II/Aggrecan
COMP: Cartilage Oligomeric Matrix Protein
COX-2: Cyclooxygenase-2
CQ-ELNs: Cissus Quadrangularis PDEVs
DGC: Density Gradient Centrifugation
DLS: Dynamic Light Scattering
DMSO: DiMethyl SulfOxide
DOX: Doxorubicin
Drp-1: Dynamin-related protein 1
ECM: Extracellular Matrix
ELISA: Enzyme-Linked Immunosorbent Assay
ENs: wintergreen-derived PDEVs
EpCAM: Epithelial Cell Adhesion Molecule
ER: Endoplasmic Reticulum
ERK: Extracellular signal-Regulated Kinase
EVs: Extracellular Vesicles
EXPO: EXocyst-Positive Organelle
FFF: Field Flow Fractionation
GelMA: Gelatin Methacryloyl
GENs: Ginseng PDEVs
GIT: GastroIntestinal Tract
GNVs: Grapefruit-derived Nanovesicles
GqDNVs: Goji berry-derived PDEVs
hUCMSCs: Human Umbilical Cord Mesenchymal Stem Cells
HBsAg: Hepatitis B Surface Antigen
HIF-1α: Hypoxia-Inducible Factor-1alpha
HO-1: Heme Oxygenase-1
HPCA: Hippocampal CD11b
HPLC: High-Performance Liquid Chromatography
HUVECs: Human Umbilical Vein Endothelial Cells
IκBα: Inhibitor of Nuclear Factor Kappa B Alpha
IGF-1: Insulin-like Growth Factor-1
IL-6: Interleukin-6
ILVs: Intraluminal Vesicles
ISEV: International Society for Extracellular Vesicles
JNK: c-Jun N-terminal kinase
Keap1: Kelch-like ECH-associated protein 1
lEVs: Large Extracellular Vesicles
miRNA: microRNA
mTOR: mammalian Target Of Rapamycin
MAPK: Mitogen-Activated Protein Kinase
Mef2D: Myocyte Enhancer Factor 2D
MoEVLPs: Morinda Officinalis-derived PDEVs
MEK1/2: MAPK/ERK kinase 1/2
MMPs: Matrix Metalloproteinases
MS: Mass Spectrometry
MSCs: Mesenchymal Stem Cells
MVB: Multivesicular Body
MWCO: Molecular Weight Cut-Off
Nrf2: Nuclear factor erythroid 2-related factor 2
NADPH: Nicotinamide Adenine Dinucleotide Phosphate
NF-κB: Nuclear Factor Kappa-Light-Chain-Enhancer of activated B cells
NFATc1: Nuclear Factor of Activated T-cells, Cytoplasmic 1
NLRP3: NOD-, LRR- and pyrin domain-containing protein 3
NOX4: NADPH Oxidase 4
NTA: Nanoparticle Tracking Analysis
NTU: NanoTized Unit (from spinach)
OA: OsteoArthritis
OATNs: Oat-derived PDEVs
OPN: Osteopontin
ORI: Oregonin I
p70S6K: Ribosomal protein S6 kinase beta-1
piRNA: Piwi-interacting RNA
PBS: Phosphate-Buffered Saline
PDEVs: Plant-derived Extracellular Vesicles
PDNVs: Plant-derived Nanovesicles
PEG: Polyethylene Glycol
PgEVs: Pomegranate-derived PDEVs
PI3K/Akt: Phosphoinositide 3-kinase/Protein Kinase B
PPAR-γ: Peroxisome Proliferator-Activated Receptor Gamma
PVA: PolyVinyl Alcohol
Rabex: Red cabbage-derived PDEVs
RANKL: Receptor Activator of Nuclear Factor Kappa-B Ligand
Runx2: Runt-related transcription factor 2
ROS: Reactive Oxygen Species
sEVs: Small Extracellular Vesicles
sRNA: small RNA
S100A4: S100 Calcium Binding Protein A4
SEC: Size Exclusion Chromatography
SDS-PAGE: SDS-polyacrylamide gel electrophoresis
SOX9: SRY-box transcription factor 9
SP-EVs: Spirulina-derived PDEVs
STS: Staurosporine
Syk: Spleen tyrosine kinase
TA: Tannic Acid
TELVs: Tomato-derived PDEVs
TEM: Transmission Electron Microscopy
TGN: trans-Golgi network
TLC: Thin-Layer Chromatography
TNF-α: Tumor Necrosis Factor-alpha
UC: Ultracentrifugation
UF: UltraFiltration
UV-MALS: Ultraviolet-Multi-Angle Light Scattering
vAW: vacuum-assisted washing
VIB: Vesicle Isolation Buffer
VSMC: Vascular Smooth Muscle Cell
YAP1: Yes-associated protein 1
