# Supplementary material for: Plant-derived extracellular vesicles in skin and bone tissue engineering: current status, challenges, and future perspectives
Source: Front Bioeng Biotechnol. 2026 Mar 19;14:1764724. doi: 10.3389/fbioe.2026.1764724 (PMC13044024; doi:10.3389/fbioe.2026.1764724)
Supplement: Supplementary file 1 [file DataSheet1.pdf]

Supplementary Material

1 Supplementary Data

Supplementary Material should be uploaded separately on submission. Please include any supplementary data, figures and/or tables.

Supplementary material is not typeset so please ensure that all information is clearly presented, the appropriate caption is included in the file and not in the manuscript, and that the style conforms to the rest of the article.

2 Supplementary Figures and Tables

For more information on Supplementary Material and for details on the different file types accepted, please see here.

2.1 Supplementary Figures

Supplementary Figure 1. The figure legends are required to have the same font as the main text, 12 point normal Times New Roman, single spaced. Please use a single paragraph for each legend and prepare the figures keeping in mind the PDF layout.

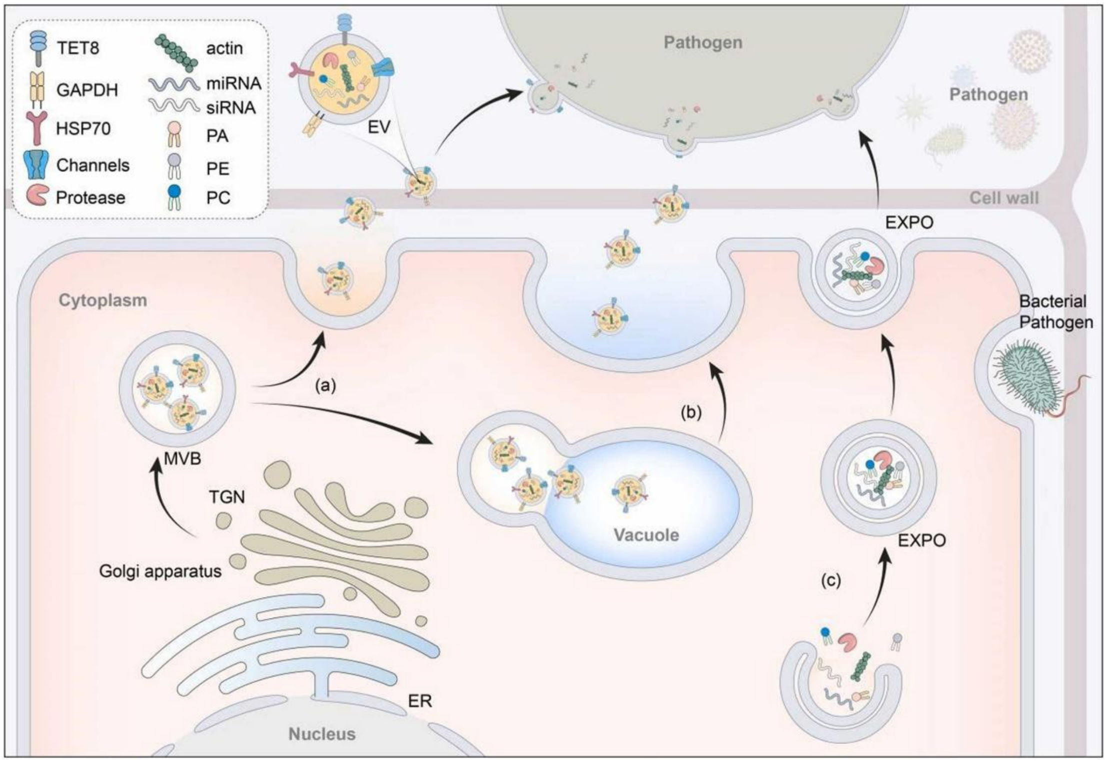

Fig. 1. Taking exosomes as an example, elucidating the basic morphology, constituents, and release mechanisms of PDEV in plants(25). Plant cells excrete EVs to counter bacterial, fungal, and other pathogenic invasions through the following pathways. Pathway (a). delineates the fusion of MVB and the plasma membrane, resulting in the release of intraluminal vesicles (ILVs) as exosomes. Pathway (b). describes the release of intraluminal EV (IEV) through the vacuolar fusion with plasma membranes. Pathway (c). shows EXPO secretion. Abbreviations: MVB, multivesicular bodies; ER, endoplasmic reticulum; TNG, trans-Golgi network; EXPO, exocyst positive organelles.

Abbreviations: MVB, multivesicular body; EXPO, exocyst-positive organelle; sRNA, small RNA; mRNA, messenger RNA.

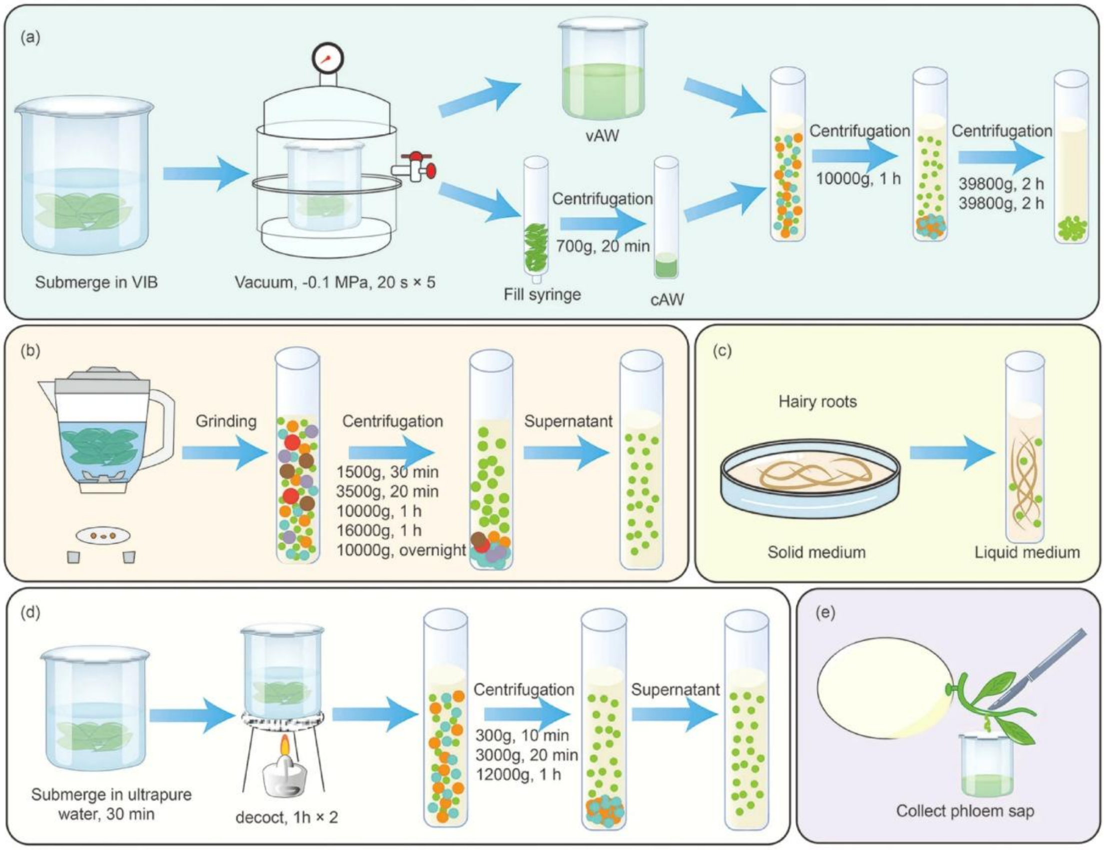

Fig. 2. Schematic illustration of common preprocessing methods for the isolation of PDEVs(58).(A) Tissue infiltration centrifugation;(B) Tissue disruption;(C) Conditioned medium method;(D) Decoction method;(E) Stem incision method.

Abbreviations: VIB, vesicle isolation buffer; vAW, vacuum-assisted washing; cAW, centrifugation-based washing.

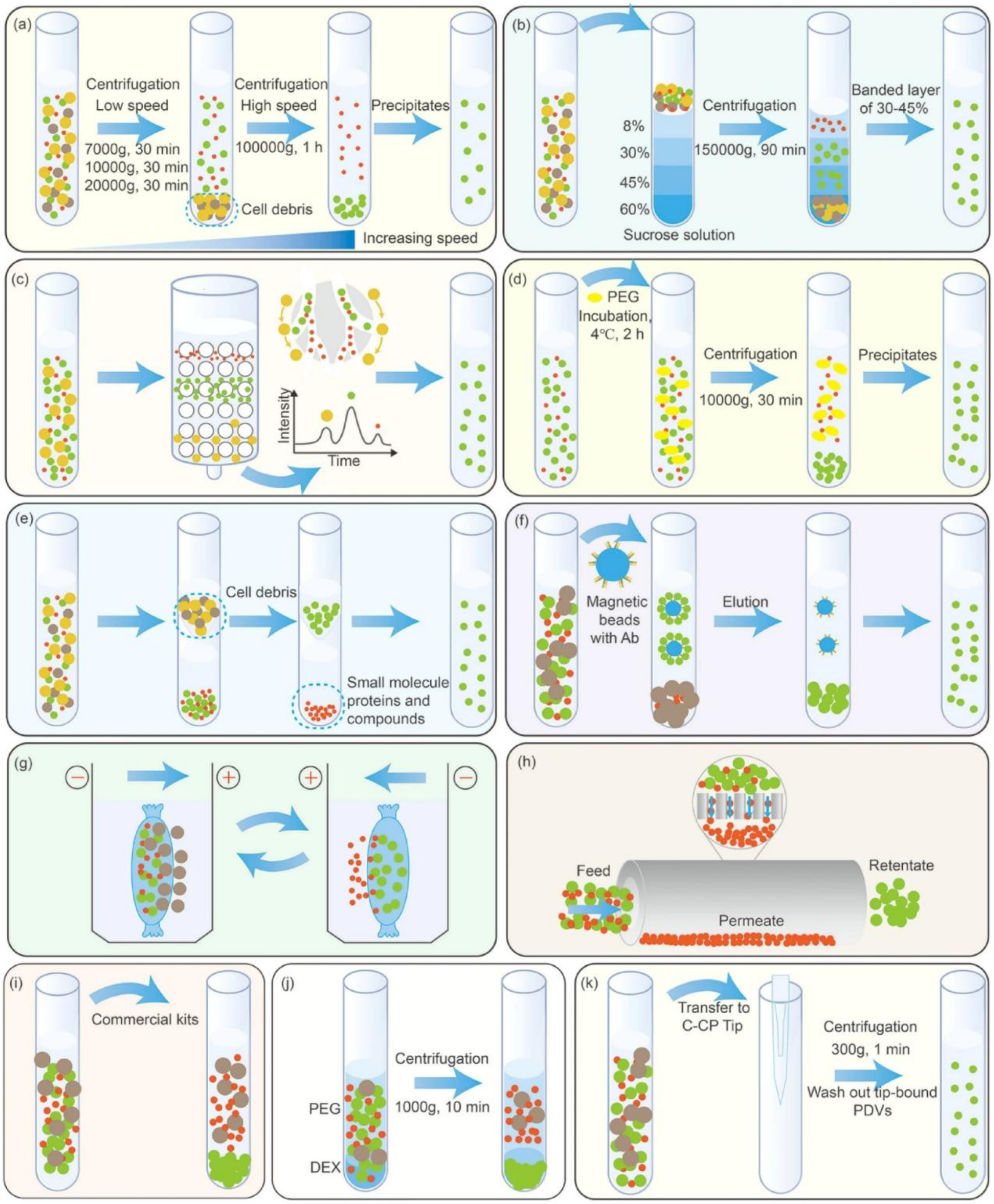

Fig. 3. Methods for the Separation of PDEVs.Illustrated are commonly employed techniques for PDEV isolation(58):(A) Differential ultracentrifugation;(B) Density gradient centrifugation;(C) Size exclusion chromatography;(D) Polyethylene glycol (PEG)-based precipitation;(E) Ultrafiltration;(F) Immunoaffinity capture;(G) Electrophoresis;(H) Tangential flow filtration;(I) Commercial kit-based isolation;(J) Aqueous two-phase separation;(K) Capillary channel polymer (C-CP) fiber spin-tip method.

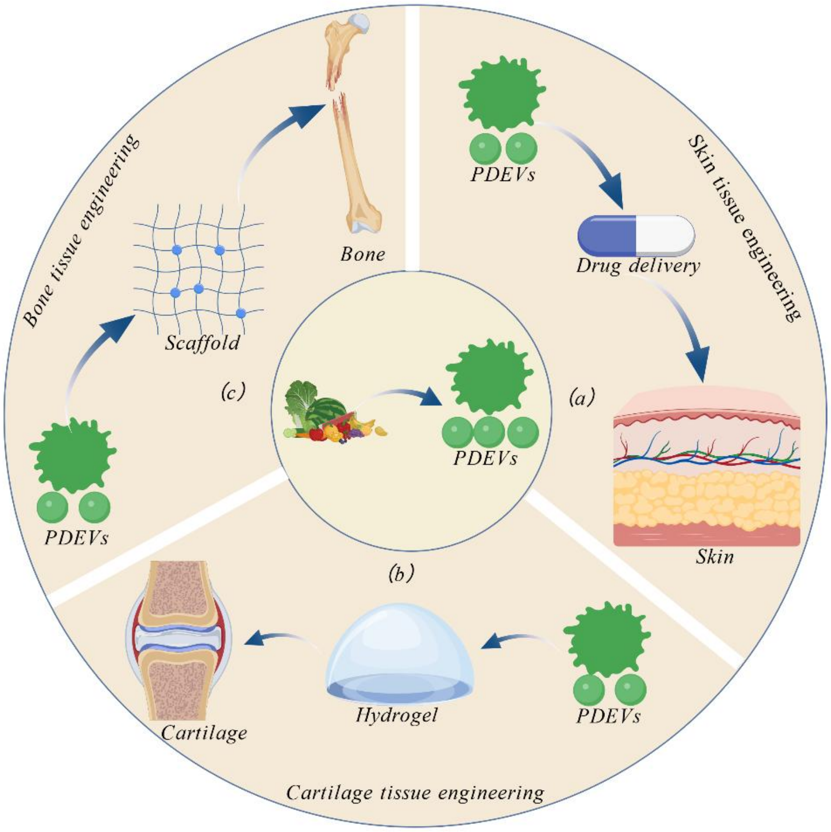

Fig. 4. PDEVs and Tissue Engineering. PDEVs serve as natural bioactive carriers. Engineered PDEVs can exert positive regulatory and therapeutic effects on (a) skin, (b) cartilage, and (c) the skeletal system.

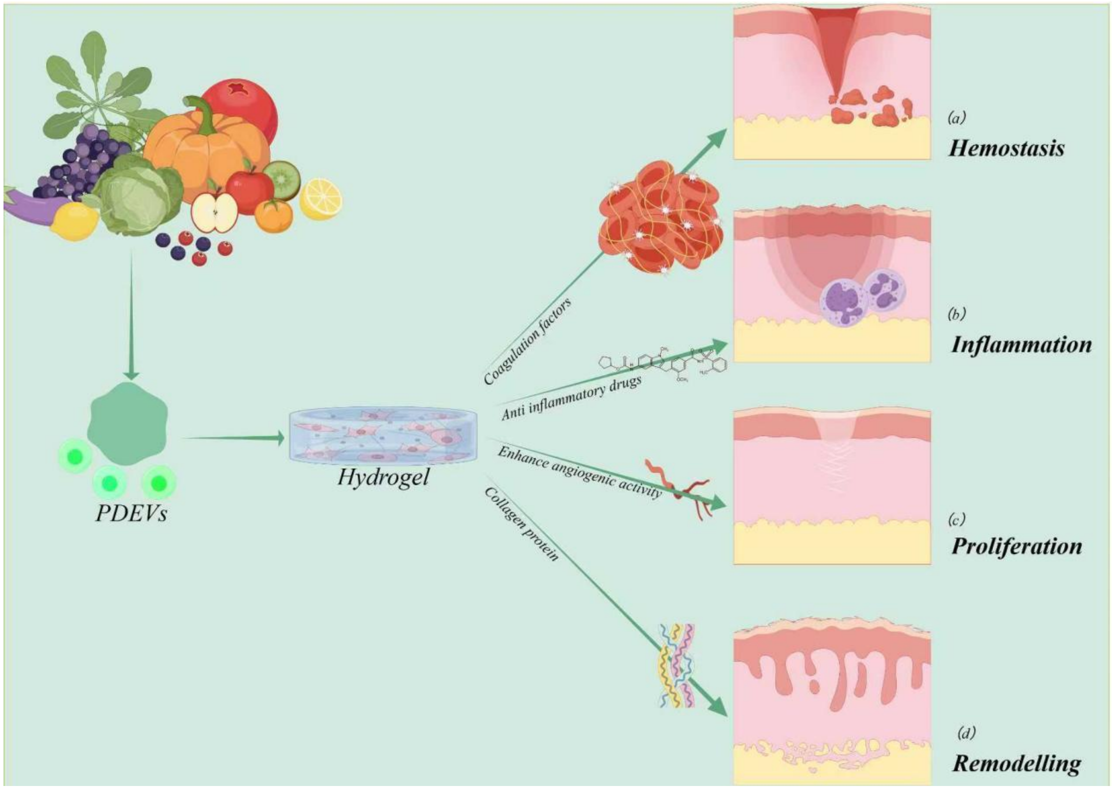

Fig. 5. PDEVs exert multifaceted effects in wound healing PDEVs simultaneously influence multiple stages of wound healing— (a) PDEVs primarily promote coagulation by interacting with blood cells, coagulation factors, and platelets; (b) PDEVs create a healing-favorable microenvironment through anti-inflammatory, antimicrobial, antioxidant, and immunomodulatory properties, while also delivering anti-inflammatory drugs to reduce wound inflammation; (c) PDEVs promote angiogenesis, enhance wound blood supply, and accelerate healing; (d) PDEVs facilitate complete wound closure with scar formation by upregulating collagen type I and remodeling collagen type III.

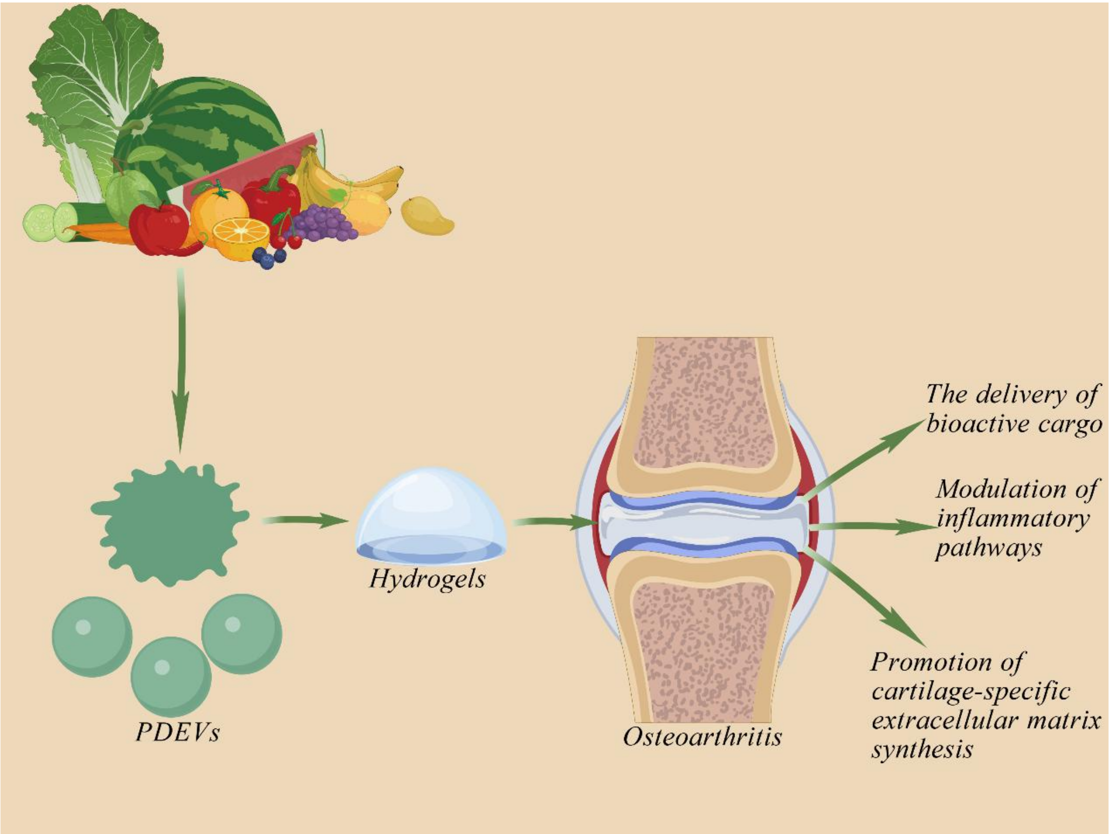

Fig. 6. Repair Mechanism of PDEV on the Cartilage System. PDEV promotes cartilage repair by delivering growth factors, modulating inflammatory responses, and stimulating the synthesis of cartilage-specific extracellular matrix.

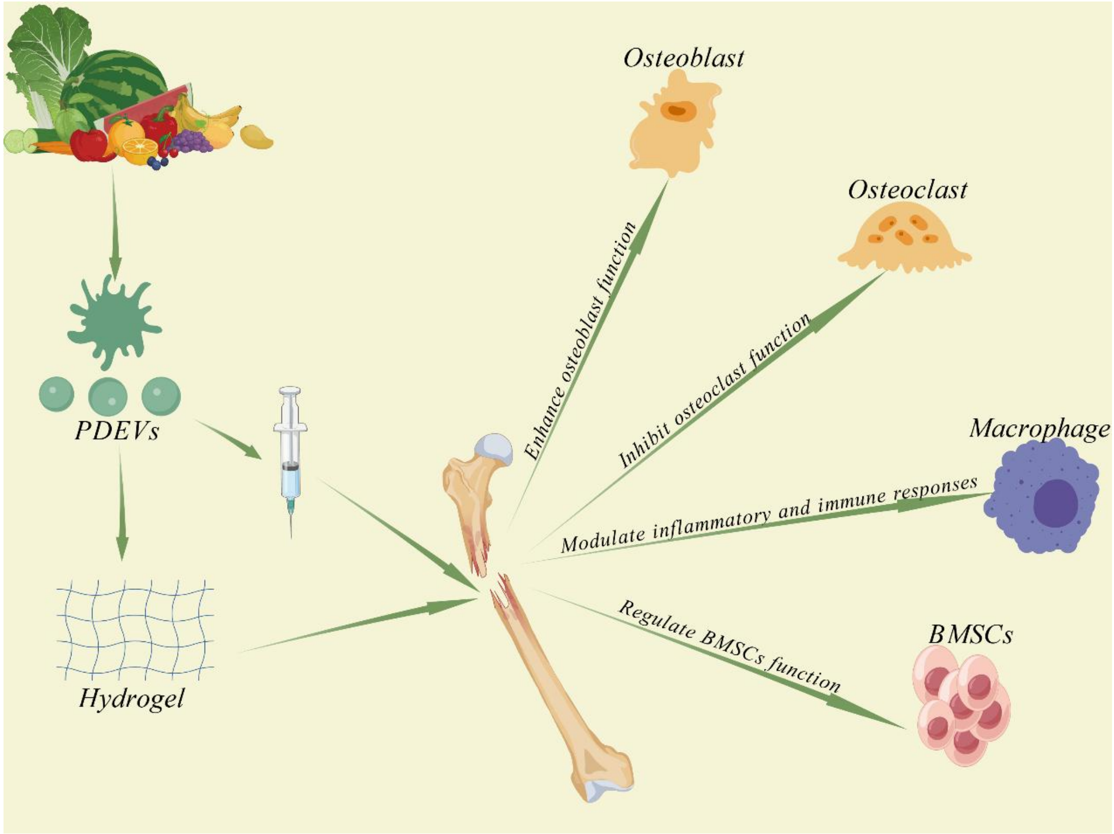

Fig. 7. Mechanism of PDEV in Fracture Healing. PDEV intervenes in fracture healing by enhancing osteoblast differentiation and mineralization, suppressing osteoclast differentiation and activity, modulating inflammatory and immune responses, and regulating the function of BMSCs.

Table 1 : Techniques for the separation and purification of PDEV and their applications.

Separation Technique	Principle	Key Parameters (Typical yield, purity, processing time)	Advantages & Disadvantages	Suitable Scenarios
Differential Centrifugation (UC)	Density	Yield: Medium; Purity: Low-Medium; Processing Time: 2–4 h (for one round)	Advantages: Handles large volumes; no requirement for specialized additives.<br>Disadvantages: Potential vesicle damage;time-consuming; equipment-intensive.	Large-scale preparation; Initial enrichment step.
Density gradient centrifugation	Precipitation Velocity and Density Magnitude	Yield: Medium; Purity: High; Processing Time: 16–20 h (including gradient formation)	Advantages: High purity and resolution; minimal mechanical damage.<br>Disadvantages: Lengthy procedure; gradient medium may contaminate	Basic research requiring high purity; Small-scale precise isolation.

(DGC)			samples.	
Ultrafiltration centrifugation (UC)	Particle Size	Yield: High; Purity: Medium; Processing Time: 1–3 h	Advantages: High efficiency; low cost; preserves native morphology. Disadvantages: Membrane fouling; protein contamination.	Medium to large-scale preparation; Rapid initial concentration.
Size exclusive chromatography (SEC)	Particle Size	Yield: Medium-High; Purity: High; Processing Time: 1–2 h (per run, column reusable)	Advantages: Good purity; gentle process; reusable columns. Disadvantages: Limited sample volume per run; dilution of samples.	Small to medium-scale preparation; Applications requiring high biological activity.
Polymer-based precipitation	Reduce the Solubility of EV in Water	Yield: High; Purity: Low; Processing Time: 4–12 h (including incubation)	Advantages: Simple; scalable; no special equipment. Disadvantages: Low purity; co-precipitation of contaminants.	Large-scale initial capture; Pre-purification for diagnostic use.
Immunoaffinity capture-based technique	Directional Enrichment of EV with Specific Covalent or Affinity Magnetic Beads	Yield: Low; Purity: Very High; Processing Time: 2–6 h (highly variable)	Advantages: Extremely high specificity and purity. Disadvantages: Low yield; high cost; limited to known markers.	Target-specific basic research; Small-scale studies requiring highly specific subpopulations.
Field flow fractionation (AF4)	A force field is applied perpendicular to the sample flow, separating particles based on the differences in diffusion coefficients between particles of varying sizes	Yield: Medium; Purity: High; Processing Time: 1–2 h	Advantages: High resolution; minimal shear stress; no stationary phase. Disadvantages: Specialized equipment; optimization required.	Basic research; Characterization and functional analysis of subsets.
ATPS	Utilizing phase systems formed by two immiscible polymers or polymer-salt solutions for partitioning separation	Yield: Medium; Purity: Medium-High; Processing Time: 3–8 h	Advantages: Gentle conditions; effective contaminant removal. Disadvantages: System optimization can be complex.	Medium-scale preparation; Purification of sensitive vesicles.
